# Attunement in Music Therapy for Young Children with Autism: Revisiting Qualities of Relationship as Mechanisms of Change

**DOI:** 10.1007/s10803-020-04448-w

**Published:** 2020-03-18

**Authors:** Karin Mössler, Wolfgang Schmid, Jörg Aßmus, Laura Fusar-Poli, Christian Gold

**Affiliations:** 1 GAMUT – The Grieg Academy Music Therapy Research Centre, NORCE Norwegian Research Center, Nygårdstangen, Postbox 22, 5838 Bergen, Norway; 2 Department of Music, GAMUT – The Grieg Academy Music Therapy Research Centre, University of Bergen, Postbox 7805, 5020 Bergen, Norway; 3 Department of Clinical and Experimental Medicine, Psychiatry Unit, University of Catania, Via Santa Sofia 78, 95123 Catania, Italy

**Keywords:** Attunement, Restricted and repetitive behavior, Sensory processing, Affect, Body, Child and parent perspectives

## Abstract

This study examined whether musical and emotional attunement predicts changes in improvisational music therapy with children with autism (4–7 years, *N* = 101, majority: no/limited speech, low IQ), assessed over 12 months. Attunement, as observed from session videos, and changes in generalized social skills, judged by blinded assessors and parents, were evaluated using standardized tools (Assessment of the Quality of Relationship, Improvisational Music Therapy Principles, ADOS, SRS). In contrast to the smaller pilot, we did not find significant effects between attunement and changes in outcomes, only tendencies in the same direction are observed. Findings suggest that symptom severity is associated with the therapist’s ability to attune to the child. They further raise questions concerning outcome selection and user involvement.

**Electronic supplementary material:**

The online version of this article (10.1007/s10803-020-04448-w) contains supplementary material, which is available to authorized users.

## Case: Car Blues with Tom

Tom was heading for his favorite place at the window bench. From there he could see all the cars on the street. He did not show interest in any of the music instruments that were available in the room. Nor, did he show interest in me. I joined him sitting on the bench and looking out of the window, fantasizing that he was fascinated by all those moving cars. Going along with my inner resonance, I started singing about what I could see on the street. A “car blues” emerged. After musically accompanying the moving cars and us watching them for a couple of sessions, I introduced a chorus line to the familiar car blues which was more dynamic and faster in tempo. I imagined that a three-year-old boy might be full of energy and might like to move himself more than he was showing when just sitting on the bench. When hearing the new chorus, Tom jumped from the bench and started spinning around. The music seemed to touch him, somehow woke him up. Immediately, I synchronized my chorus line with his movements, trying to match his pulse and attuning to his dynamic. When exactly meeting his movement with my music, Tom stopped spinning around for some seconds, looked and smiled at me for the first time. (Experience from a music therapy session with “Tom” and the first author.)

Within infancy, sensitive attunement between the infant and the primary caregiver creates and shapes relational experiences leading to modes of attachment (Ainsworth et al. 1978; Bowlby 1969; Meins et al. 2001) and further development of social understanding (Stern 1985/2000). Attunement becomes observable through synchronized body movements as well as empathetically coordinated actions, accompanied by affective regulation, sharing and expressions. Through the human capability to detect cross-modal correspondences (Meltzoff 1990) infants are able to synchronize their movements in time, form and intensity with their caregiver and to share inner subjective states with them (Stern 1985/2000; Trevarthen 1998). Successful mutual affective interactions between infant and caregivers, powered by the ability to progress between states of attunement, mis-attunement and re-attunement (Tronick and Cohn 1989), supports the infants’ ability to organize and self-regulate sensorimotor and affective experiences (Stern 1985/2000; Trevarthen and Aitken 2001). At the same time, it strengthens and extends the caregivers` capabilities to affectively engage with the child. Through mutual attunement processes, the infants learn about their agency, develop motivation for co-regulation and gain confidence in mental closeness (Tronick and Beeghly 2011). All of these early achievements promote further social, cognitive, and language development (Greenspan and Shanker 2007). When the infant discovers that the focus of attention, the feeling state, and intention can be shared (Stern 1985/2000) and mismatching affective states can be repaired (Tronick 2008), intersubjectivity can naturally unfold.

However, these experiences might not necessarily evolve in infants later diagnosed with an autism spectrum condition (ASC). First-hand accounts presenting lived experiences with autism depict challenges in perceiving, sensing, moving, and emoting all of which form and inform the abilities to communicate, relate, and participate in life (Robledo et al. 2012). Deficits in social communication and interaction are well described as core symptoms in ASC (American Psychiatric Association 2013) and, corresponding with the lived experiences of people with ASC, are found to be correlated with deficits in sensory processing (Gliga et al. 2014; Thye et al. 2018) and emotion dysregulation (Samson et al. 2014) in recent research. Moreover, deficits in cognition and communication have been proposed to be secondary to weaknesses in prospective control of movements and the maturation of sensory-motor skills interfering with the ability to affectively engage in interactions (Delafield-Butt and Trevarthen 2017; Muratori and Maestro 2007; Trevarthen and Delafield-Butt 2013). Intersubjectivity relies on the “precise timing and rhythms of two motor systems operating in time and in tune with one another, generating a ‘communicative musicality’ (Malloch and Trevarthen 2009) in mutual acts” (Delafield-Butt and Trevarthen 2017, p. 129). This sensory-motor timing is crucial to social understanding, participatory sense-making and the natural growth into family rhythms and patterns, however appears to be disrupted in people with ASC (De Jaegher 2013; Delafield-Butt and Trevarthen 2017). Differences in organizing, synchronizing, and regulating sensory information and movement belong to the lived experiences of people with ASC. These differences might hamper or prevent development but, more importantly, these differences need to be seen and acknowledged as they can incorporate the persons unique ways of communicating and engaging with the world (Donnellan et al. 2012).

Hence, it has been suggested that enhanced focus should be given to the ways children with ASC sense, move, sound, and emote as resources for expression, communication, and participatory sense-making (De Jaegher 2013). Therapeutic approaches showing awareness towards sensory processing, movement and motor control, synchronization in motion and emotion as well as affect regulation are likely to create opportunities for developing a shared narrative and to prompt further development of social interaction skills in children with ASC (Trevarthen and Delafield-Butt 2013).

## Musical and Emotional Attunement

In music therapy, musical and emotional attunement is used to allow for moments of synchronization, to work on sensory integration and affect regulation, and to create moments of affective attunement, emotional sharing, and hence an emerging shared narrative. Successful attunement is assumed to increase opportunities for the child to improve self-awareness, to experience shared attention and social reciprocity, and to enhance communication (Geretsegger et al. 2015; Schumacher et al. 2019). However, little is known about whether attunement can serve as a mechanism of change in therapeutic settings with people with ASC. Synchronization might represent one promising feature of attunement that can be associated with positive development. Autism research has shown that synchronization of interpersonal behavior predicts socially engaged imitation in toddlers (Landa et al. 2011). Movement synchronization and imitation was associated with increased empathy in adults (Koehne et al. 2016a, b) as well as increased emotion inference (Koehne et al. 2016a, b). A pilot investigation to the present study, incorporating a more broad understanding of attunement including synchronization, has shown that the therapist’s ability to attune musically and emotionally to the child’s expressions, might be an important predictor of the development of social skills in preschool children with ASC (Mössler et al. 2019).

As depicted in the opening case vignette, music can be related to observable behavior (e.g. watching cars), a sensed invisible inner world (fascinated by things moving), body movements or sounds (spinning around), and the child’s affective dynamic (from calm to excited and joyful). Similarly to primary caregivers when interacting with their infants, music therapists can utilize the pre-linguistic musical features of time (rhythm), form (sound), and intensity (dynamic) to create attunement, supporting coherences between sensory and affective modalities within the child (intra-personal) as well as between the child and the therapist (inter-personal) (Schumacher et al. 2019). Since attunement seems to be a pivotal relational factor within human development shaping our social abilities and understanding, the present study aims to investigate further whether attunement embodies a mechanism of change when working on the development of social interaction skills in music therapy with preschool children diagnosed with ASC. Based on promising results of the pilot to this study (Mössler et al. 2019), we aim to replicate these results by extending the previous sample size. The research questions to be addressed are:

- Whether the musical and emotional attunement, seen as crucial relational qualities between the music therapist and the child, predicts generalized changes in core autism spectrum traits such as limited communication and social interaction skills.
- Whether the therapy intensity, i.e. the number of music therapy sessions per week, is associated with the level of attunement achieved.

## Methods

### Study Design

The design of this study matches closely that of our previous predictor study (Mössler et al. 2019). The data were collected as part of an international randomized controlled trial which examined the effects of music therapy with children with ASC (TIME-A; Bieleninik et al. 2017a, b; ISRCTN78923965), but only the experimental arm from this trial was used for this predictor study as we were interested in process variables, namely musical and emotional attunement, predicting the effect of music therapy with children with autism. In this sense, this predictor study is best described as prospective, observational, and longitudinal because the predictors were process variables which were observed rather than experimentally assigned. The standard care arm from the original trial was excluded because the process variables do not exist there. However, participants were randomized to two intensity levels of music therapy (low-intensity versus high-intensity) in a 1:1 ratio. Randomization to these conditions was administered centrally and concealed from clinicians conducting outcome assessments (Bieleninik et al. 2017a, b). Video data used for this study were collected and analyzed as part of an extended treatment fidelity analysis of the TIME-A trial. Treatment fidelity analysis were planned and conducted for all countries participating in the TIME-A project. Ethics approval was obtained from the responsible authorities at all participating sites. Parents or guardians of participating children provided written informed consent.

### Participants

Eligible participants were between 4 and 7 years of age and diagnosed with an autism spectrum disorder according to ICD-10, confirmed with the Autism Diagnostic Observation Schedule (ADOS) (Lord et al. 2000) and the Autism Diagnostic Interview-Revised (ADI-R) (Lord et al. 1994,1993; Risi et al. 2006). Exclusion criteria defined for the overall trial were serious sensory disorders such as blindness or deafness and having received music therapy in the last 12 months. Further inclusion criteria for the present study were defined by the completeness of the data collection, specifically the number of completed music therapy sessions (at least 10) and completed outcome assessments at 5 and 12 months. In addition, sufficient video data in terms of quanitity and quality must have been accessible. Of 182 participants who were originally randomized to one of the music therapy conditions in the overall trial, 101 participants fulfilled these inclusion criteria. Thirty-seven participants were excluded due to incomplete data collection (missing music therapy sessions and/or missing outcome assessments) and 54 were excluded due to insufficient quality or availability of video data. These two categories are overlapping for 10 participants.

The sample included the 48 who were analyzed in our previous predictor study. Participants were from all nine participating countries (Australia 16, Austria 7, Brazil 1, Israel 6, Italy 11, Korea 8, Norway 5, UK 30, and USA 17). All participants from Brazil and the UK were new in this sample as these countries started recruiting later. Recruiting procedures were following the same protocol in all countries (Geretsegger et al. 2012).

### Interventions

Of the 101 participants included in this study, 50 were randomized to low-intensity and 51 to high-intensity music therapy. Low-intensity music therapy consisted of up to one session and high-intensity music therapy of up to three sessions per week, both over five months. Actual numbers of sessions were lower, on average 16.1 (SD 2.4) and 35.4 (SD 10.0), respectively. Children in the high intensity group were often not able to attend three times a week due to different reasons (e.g. sickness, other family related issues, long travel distances). Additionally, all children received usual care as locally available, and all parents/caregivers were offered three sessions of parent counselling. Within the sample of this study, all parents used these sessions as offered.

Music therapy was provided on an improvisational basis informed by developmental psychology (Geretsegger et al. 2012) and based on treatment guidelines defining common characteristics and principles of improvisational approaches in music therapy for children with ASC, hereinafter referred as improvisational music therapy (IMT) principles. IMT principles allowed for cultural sensitivity, as they could be tailored to the various contexts and cultures of the overall trial (Geretsegger et al. 2015). They were used to train music therapists in the international trial and to assess treatment fidelity. Twenty-seven qualified music therapists (19 females; age, range 23 to 55 years, M = 34.5 years; years of experience as music therapists, range 0 to 18 years, M = 6.8 years) were involved in the study, conducting music therapy sessions. Even though therapists in this trial had different training backgrounds and were coming from different cultures, treatment fidelity ratings were adequate, meaning that the therapists provided IMT on a common methodological understanding and in similar ways (Bieleninik et al. 2017a, b).

### Assessment: Process Variables

For assessing the musical and emotional attunement between the therapist and the client we used two approaches. One included an in-depth analysis using the Assessment of the Quality of Relationship (AQR). The second procedure used a more simplified approach based on the existing treatment fidelity variables for IMT that was used in the overall trial (Bieleninik et al. 2017a, b; Geretsegger et al. 2015).

The *AQR* is an expert rating tool based on developmental psychology (Schumacher et al. 2013, 2019). It assesses the child’s availability for contact and ability to engage in interactive communication on a non-verbal level, focusing on bodily and musical expressions. At the same time, it assesses the therapist’s working modes used to musically and emotionally attune to the child’s expressive and relational resources as well as needs. For rating the child’s way of relating within a music therapy session, an assessor can choose between three scales, depending on the child’s manner of expression: instrumental expression, vocal or pre-speech expression, and physical-emotional expression (reflecting the child’s body language). In addition, a fourth scale is used for assessing the therapist’s interventions and to determine whether they were attuned to the child. Each scale consists of the following seven relational qualities called “modi”: modus 0 = lack of contact/contact refusal; modus 1 = sensory contact/contact-reaction; modus 2 = functionalized contact; modus 3 = contact to oneself/self awareness; modus 4 = contact to another/intersubjectivity; modus 5 = relationship to another/interactivity; modus 6 = joint experience/interaffectivity (Schumacher et al. 2019).

When rating participants in the present study, the assessors had to choose a “main modus” in case of shifting modi within the particular sequence, representing the child’s expression and behavior best in terms of intensity and duration. Successful attunement was assumed when both child and therapist acted on the same relational modus, hereinafter referred to as *AQR match* which was rated as 1 = match or 0 = mismatch.

AQR assessments were done as consensus ratings of three trained and certified AQR assessors. Members of the assessor group included the developers of the AQR instrument as well as other music therapy practitioners and researchers who had both the compulsory AQR training and extensive experience with the instrument as well as with the client population.

AQR modi were assumed ordinal because they are related to subsequent developmental stages. For analyses, *AQR match rate* was defined as the proportion of all sequences of a given participant where a match occurred. Interrater reliability of the AQR was tested previously (Schumacher et al. 2006).

Conceptually related to the AQR match rate, we used *treatment fidelity ratings* of the overall trial (Bieleninik et al. 2017a, b; Geretsegger et al. 2015). Altogether, fidelity ratings were adequate (Bieleninik et al. 2017a, b), but here we were interested in the variation in fidelity. In addition to the total fidelity score, a sum score of eight Likert-scaled items, we were specifically interested in item 1, which asked about “musical and emotional attunement” (Geretsegger et al. 2015). This item was defined as a unique and essential principle in terms of its relevance to music therapy as it asks about how well the therapist is musically and emotionally attuned to the child. While Item 1 asks specifically about attunement, the whole scale is a somewhat broader but related measure of the therapist’s ability to match their actions and attitudes to the child’s activities and arousal levels. For example, other items ask about building a positive relationship, providing a secure environment, or following the child’s lead*.* The total score had a possible range of 0 to 40, and item 1 had a possible range of 0 to 5, representing a combination of adherence (0–3) and competence (4–5), with higher scores indicating better fidelity or better attunement, respectively (Geretsegger et al. 2015). These ratings were based on the average of two trained raters (Bieleninik et al. 2017a, b). Data were utilized from the treatment fidelity subsample of the overall trial and were available for 60 of the 101 participants in the present sample.

All process variables were rated based on randomly selected video sequences. Ten sequences of three minutes duration were selected for each child. When possible, sequences were chosen from every uneven week number (i.e., week 1, 3, 5,…19 of each therapy process); when these were not available, even week numbers (2, 4, 6,…20) were selected. Raters were blinded to the position of each sequence within the therapy process (sequences were labelled with alphabetic letters) to avoid a bias towards more positive ratings in later sessions (further details of the selection process, see Mössler et al. 2019).

### Assessment: Outcome Variables

As in the previous study, core symptoms of ASD were measured using two commonly used measures. The Autism Diagnostic Observation Schedule (ADOS; Gotham et al. 2007) was completed by independent, blinded raters, and the Social Responsiveness Scale (Constantino et al. 2003) was completed by parents; all at baseline and 5, and 12 months later (Bieleninik et al. 2017a, b; Geretsegger et al. 2012).

The ADOS is an observation instrument that is commonly used for diagnostic purposes but also to measure outcomes. Depending on language abilities and age, one of three modules and one of five scoring algorithms was chosen. Adaptations to improve sensitivity to change and consistency across time points were described previously (Bieleninik et al. 2017a, b). The ADOS Social Affect score was chosen as the primary outcome; possible scores ranged from 0 to 27 in ADOS module 3 and 0–24 in ADOS module 1 and 2, with higher scores indicating higher severity. Additionally, the ADOS total score and ADOS Restricted and Repetitive Behavior score were analyzed.

The SRS, a parent-rated scale, consists of 65 items ranging from 0 to 3, with a total sum score ranging from 0 to 195. Higher scores indicate higher autism severity.

### Statistical Analyses

Descriptive and graphical analyses were used to analyze the frequency distributions of AQR modi and their development over time.

Linear mixed-effects models (LMEs) were used to analyze whether AQR match rate predicted outcomes. We calculated both unadjusted LMEs containing only the predictors of interest, and LMEs adjusted for diagnosis, intensity of IMT (fixed effects), site, and therapist (random effects). In a sensitivity analysis, we excluded the smallest site with only one participant (Brazil) from the analysis. We restricted these analyses to AQR match rate and did not analyze AQR modi and their development over time through LMEs because linearity could not be assumed.

In exploratory analyses concerning IMT treatment fidelity scores, we calculated the same LMEs as for AQR match rate. We also analyzed correlation between these different process variables. The general significance level was set to 0.05. Since the LME models included three independent variables (main effect and to interactions), we used a Bonferroni adjustment for each model as we had to take into account multiple testing effects. This led to a marginal level of 0.0167. Calculations were done using R 3.5.1 including the nlme package. Figures were created using MatLab 9.0.

## Results

### Sample Characteristics

Baseline characteristics (Table 1) were similar between conditions, with a large majority being male and diagnosed with childhood autism (ICD-10 code: F84.0). Low average IQ, a substantial proportion with IQ < 70, and a large proportion where ADOS Module 1 was used, indicate a functioning level that is varied but includes many at the lower end of the spectrum. In addition, the participants newly added to the sample (n = 53) scored significantly higher (p = 0.024) on the ADOS restricted and repetitive behavior (RRB) domain at baseline compared to our previous sample (n = 48; mean difference = 0.9; 95% CI (0.1–1.7).

**Table 1:** Baseline characteristics of the included sample

Characteristics	All patients		High-intensity MT 3 ×/week		Low-intensity MT 1 ×/week	
	N	Value				
			N	Value	N	Value
Age^a^	101	5.4 (5.3, 5.6)	51	5.4 (5.2, 5.7)	50	5.4 (5.2, 5.6)
Sex (m)^b^	101	85 (84%)	51	43 (84%)	50	42 (84%)
Diagnosis (ICD-10)^b^	101		51		50	
Asperger's syndrome (F84.5)		4 (4%)		1 (2%)		3 (6%)
Childhood autism (F84.0)		83 (82%)		44 (86%)		39 (78%)
Pervasive developmental disorder unspecified (F84.9)		14 (14%)		6 (12%)		8 (16%)
IQ, standardized test^a^	57	78.8 (72.4, 85.3)	28	79.6 (68.6, 90.6)	29	78.1 (70.9, 85.3)
Intellectual disability (IQ < 70)^b^	97	40 (41%)	49	29 (43%)	48	19 (40%)
ADOS module^b^	101		51		50	
Module 1		61 (60%)		33 (65%)		28 (56%)
Module 2		39 (39%)		18 (35%)		21 (42%)
Module 3		1 (1%)		0 (0%)		1 (2%)
ADOS^a^						
Total score	101	18.3 (17.3, 19.3)	51	18.8 (17.4, 20.2)	50	17.8 (16.2, 19.3)
Social affect	101	14.4 (13.5, 15.2)	51	15 (13.7, 16.2)	50	13.8 (12.6, 14.9)
Language and communication	101	3.5 (3.2, 3.8)	51	3.7 (3.2, 4.1)	50	3.2 (2.8, 3.7)
Reciprocal social interaction	101	10.9 (10.2, 11.6)	51	11.3 (10.3, 12.3)	50	10.5 (9.6, 11.5)
Restricted and repetitive behavior	101	3.9 (3.5, 4.3)	51	3.8 (3.3, 4.4)	50	4 (3.4, 4.6)
SRS^a^						
Total score	101	96.1 (90.5, 101.7)	51	94.5 (87.5, 101.6)	50	97.6 (88.9, 106.3)
Awareness	101	12.2 (11.4, 13)	51	12 (11, 13)	50	12.4 (11.2, 13.7)
Cognition	101	18.2 (17, 19.4)	51	18 (16.5, 19.5)	50	18.4 (16.5, 20.4)
Communication	101	32.1 (30.1, 34.1)	51	31.6 (29, 34.3)	50	32.6 (29.4, 35.7)
Motivation	101	15.1 (14.1, 16.2)	51	14.9 (13.5, 16.3)	50	15.4 (13.8, 16.9)
Mannerisms	101	18.4 (17, 19.9)	51	18.1 (16.2, 20)	50	18.8 (16.7, 20.9)

Diagnostic codes are according to ICD-10

^a^Mean (SD)

^b^N (%)

*MT* music therapy, *IQ* intelligence quotient, *ADOS* autism diagnostic observation schedule, *SRS* social responsiveness scale

### Descriptive Analysis of AQR Modi

**Fig. 1:**
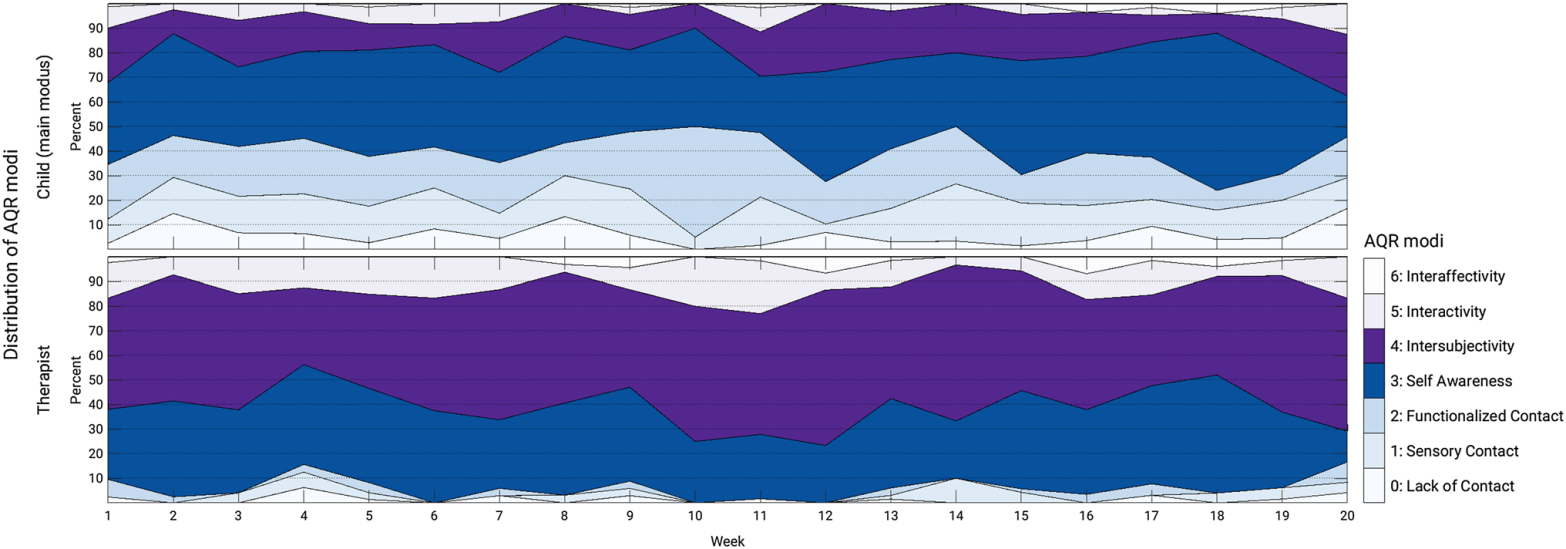
Distribution of the child’s and the therapist’s AQR modi over the course of therapy. *AQR* assessment of the quality of relationship

**Fig. 2:**
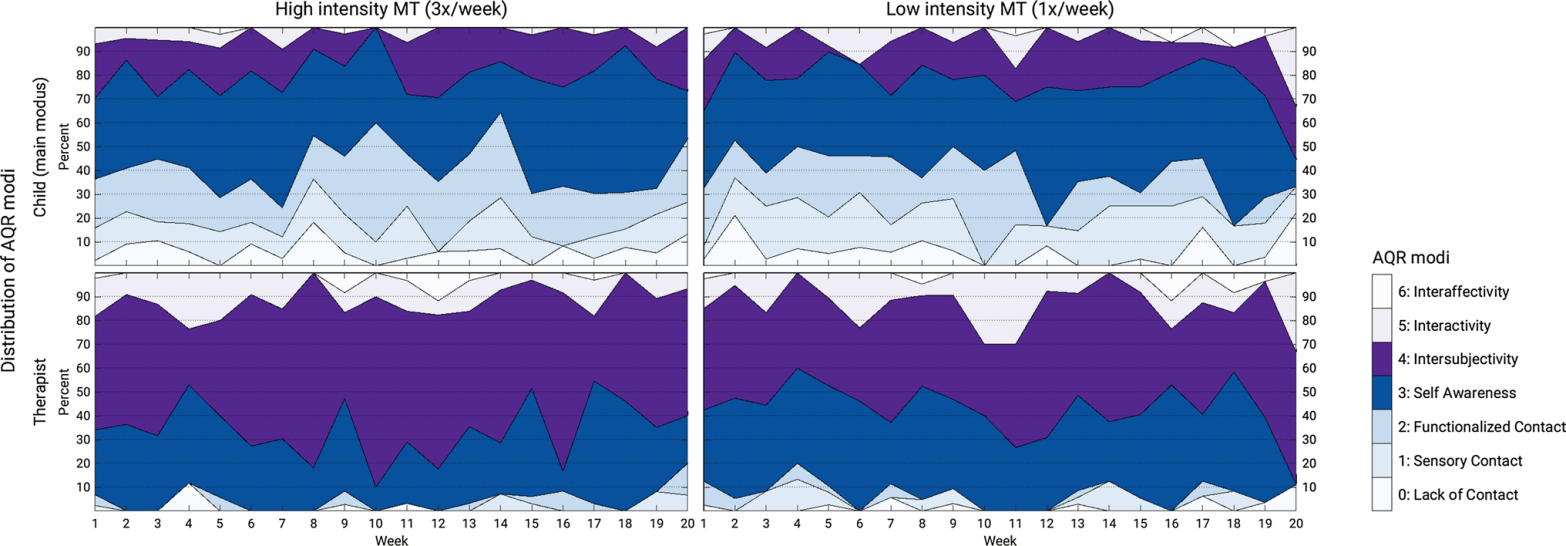
Distribution of the child’s and the therapist’s AQR modi over the course of therapy within the two intervention groups. *AQR* assessment of the quality of relationship

Graphical analysis (Fig. 1) indicates that the *child’s main modus* and the *therapist’s modus* remain relatively stable over the 20 weeks of therapy. The most common AQR modus of the child, modus 3 (self-awareness, dark blue area in Fig. 1), was observed in about 30–40% of therapy sessions in each week. This was followed by modus 2 (functionalized contact), modus 4 (intersubjectivity), and modus 1 (sensory contact). Modus 3 was also observed in 20–40% of therapy sessions for the therapist. In contrast to the modus distribution of the child, the most common modus for the therapist was modus 4 (intersubjectivity, 40–50% in each week; purple area in Fig. 1). Looking at the two intervention groups, we can see a similar picture (Fig. 2) and there is no significant difference in the match rates (p = 0.329). Both children randomly assigned to high- and low-intensity music therapy were typically assessed with modus 3, followed by modus 2, 4, and 1. For the therapist we might be able to see a tendency that modus 4 was observed even more frequently (40–60% in each week) for therapists working with children randomly assigned to the high-intensity group.

These findings might indicate that chances for therapist and child to meet on the same modus may be highest when the child is functioning on relatively higher modi (3–5), whereas children who present on one of the lower modi (1–2) may not be met by their therapist on that same modus. The lowest (0) and highest modus (6) were of marginal relevance, although there was some occurrence (up to 10%) of the lowest modus in the child.

**Fig. 3:**
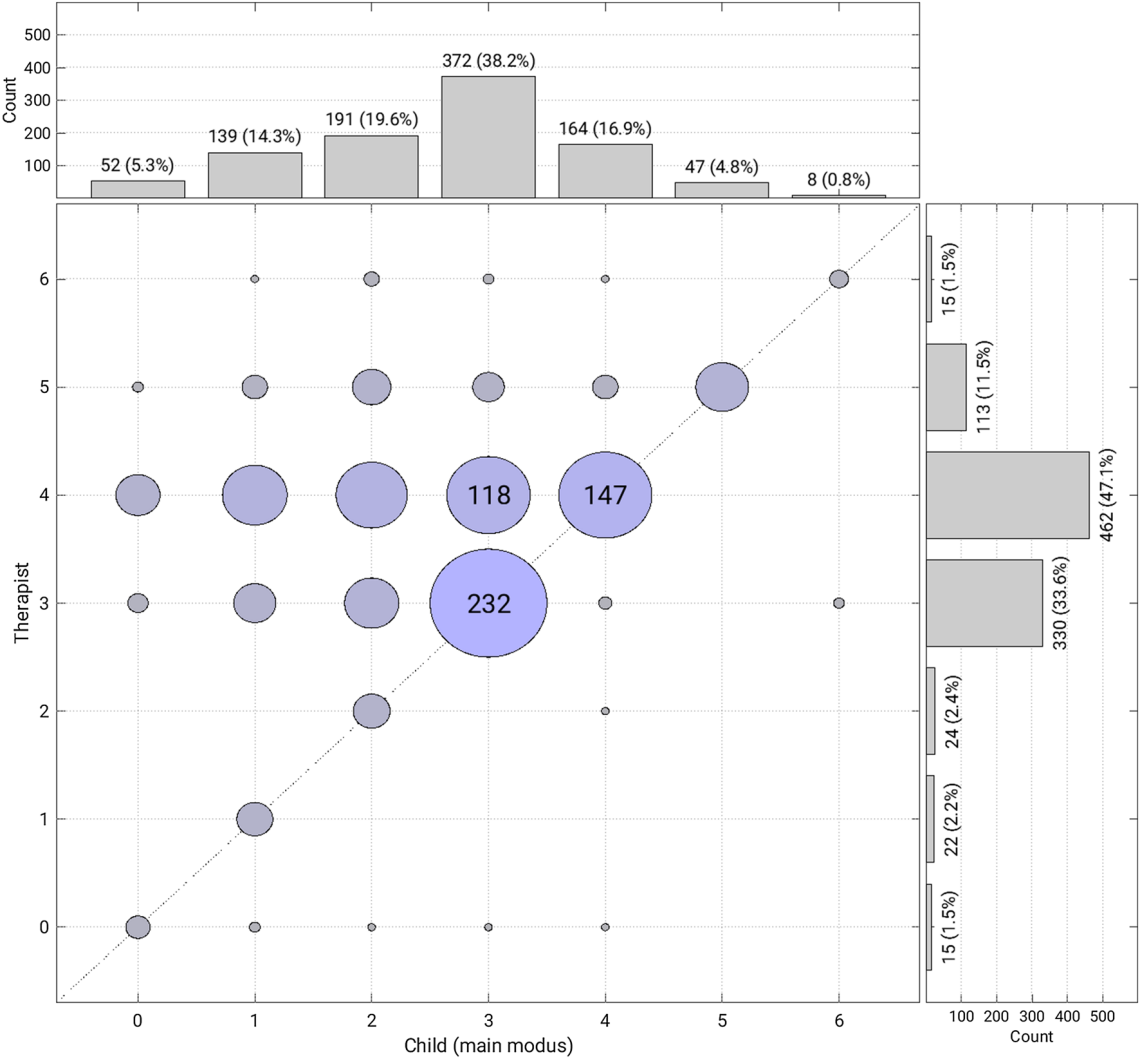
Joint frequency distribution of the child’s and the therapist’s AQR modi. Bubbles indicate frequency of occurrence of each combination of AQR modi across all weeks; the area of the bubbles is proportional to the number of sequences. For the highest frequencies, the number is shown within the bubble

The graphical analysis of the joint frequency distribution of the child’s main modus and therapist’s modus (Fig. 3) also indicates the typical direction of mismatch. Matches of the same modus (bubbles on the diagonal line) were most common when both were on modus 3 (232 sequences) or modus 4 (147 sequences). When child and therapist were on different modi, the therapist was typically on a higher modus than the child (bubbles above the diagonal, Fig. 3), whereas the opposite situation (below the diagonal) was very rare.

An analysis by week showed similar patterns (Online Appendix 1). A match was seen in 493 (49.6%) of 994 sequences. The match rate differs significantly between the three AQR scales for the child (p < 0.001). Matches on the child’s main modi were most frequently observed for the vocal scale (69.7%; 53 matches out of 76 observations on this scale), followed by a 53% match rate on the instrumental scale (298 matches out of 561 observations) and a 39,8% match rate on the physical-emotional scale (142 matches out of 357 observations). According to these findings, we might assume that attunement is achieved more easily when the child’s main modus of expression is vocal or instrumental play. Based on these findings, it seems to be harder to relate to children mainly communicating and emoting via bodily expressions.

### Linear Mixed-Effects Models

The results of the LMEs for the main variables (Table 2) showed no significant effects between the AQR match rate and changes in ADOS and SRS. However, similar to our previous smaller pilot we can still observe a significant time independent main effect of AQR match rates, indicating that a higher AQR match rate was associated with lower scores in ADOS Total (p < 0.0001) and ADOS subscales (Social Affect, p = 0.0001; Restricted and Repetitive Behavior, p = 0.0115) at baseline.

**Table 2:** Linear mixed effects (LME) models to predict generalized outcomes from the AQR match rate

	B	95%CI	df	t	p-value
ADOS total					
AQR match rate	− 9.24	(− 13.30, − 5.18)	99	− 4.47	< .0001
AQR match rate × (5 months vs. BL)	0.44	(− 2.61, 3.49)	194	0.28	0.7779
AQR match rate × (12 months vs. BL)	− 0.62	(− 3.73, 2.48)	194	− 0.39	0.6966
ADOS social affect					
AQR match rate	− 7.21	(− 10.59, − 3.83)	99	− 4.19	0.0001
AQR match rate × (5 months vs. BL)	0.40	(− 2.23, 3.03)	194	0.30	0.7673
AQR match rate × (12 months vs. BL)	− 0.36	(− 3.04, 2.32)	194	− 0.26	0.7915
ADOS restricted and repetitive behavior					
AQR match rate	− 2.03	(− 3.59, − 0.48)	99	− 2.58	0.0115
AQR match rate × (5 months vs. BL)	0.04	(− 1.43, 1.52)	194	0.06	0.9555
AQR match rate × (12 months vs. BL)	− 0.26	(− 1.76, 1.24)	194	− 0.34	0.7331
SRS total					
AQR match rate	− 13.49	(− 36.28, 9.31)	99	− 1.16	0.2483
AQR match rate × (5 months vs. BL)	− 16.06	(− 31.85, − 0.27)	169	− 1.99	0.0486
AQR match rate × (12 months vs. BL)	− 3.24	(− 20.03, 13.56)	169	− 0.38	0.7072

All calculations based on linear mixed effects models

*ADOS* Autism diagnostic observation schedule, *AQR* assessment of the quality of relationship, *BL* baseline, *MT* music therapy, *SRS* social responsiveness scale

These results were not altered in the sensitivity analysis excluding Brazil. Furthermore, there was no significant difference in AQR match rates between high-intensity and low-intensity music therapy (p = 0.329). No differences were found between the adjusted and unadjusted model when applying a Bonferroni adjusted significance level for all outcomes (see Online Appendix 2).

Treatment fidelity ratings of IMT principles were positively correlated with AQR match rate (total IMT fidelity score: r = 0.55, 95% CI 0.34 to 0.70; IMT item 1: r = 0.49, 95% CI 0.27 to 0.66). LMEs using IMT principles as predictors did not show any significant relationship with clinical outcomes (see Online Appendix 3).

## Discussion

### Findings

Descriptive statistics of AQR modi and match rates: Therapists tend to be on a higher modus than children, they match on not more than 50%. This confirms the findings from our previous smaller study (Mössler et al. 2019). Additionally, this study showed that the match rate changes little over time; later sessions showed similar match rate as earlier ones. Furthermore, this study showed that when a match occurs, it occurs on one of the higher modi (3 and 4). Children were often assessed with modus 2 (20%, see upper panel Fig. 3), but therapists were observed on modus 2 only in 2% of all incidents (see right-hand panel of Fig. 3). A similar picture can be described for modus 1 (child: 14%, therapist: 2%). It may be that therapists are either not aware of these lower modi or not trained to meet children on these modi. However, the therapists in the study were not specifically trained in the use of the AQR, and not all were necessarily intending to work according to these principles. All therapists did however work according to the IMT principles (Geretsegger et al. 2015), which this study showed to be highly correlated with AQR match. The high correlation between IMT principles (total fidelity scale and item 1 on musical and emotional attunement) and the AQR match rate is not surprising because both scales were derived from the same concepts of child development and mother-infant communication (Stern 1985/2000). However, since the ratings came from different raters, it is still a confirmation of the relevance and validity of both scales. As rating IMT principles is simpler and does not require specialized AQR training, they may also serve as a useful substitute when AQR raters are not available.

Further, the main hypothesis of the present study, concerning the prediction of clinical outcomes, could not be confirmed. Neither AQR match rate nor IMT item 1 predicted changes in autism severity or other outcomes. This is in contrast to the previous smaller study (Mössler et al. 2019), which suggested that effects of IMT were better when match rates were higher. This could be due to clinical or cultural differences, but most likely due to chance.

Similar to our smaller pilot, in which we applied a conventional significance level (p < 0.05), we could replicate the effect between attunement and changes in SRS total (p = 0.0486; see Table 2 and Mössler et al. 2019) at 5 months, supplemented by an interaction effect on the SRS cognition subscale (p = 0.0478; see Online Appendix 2) in this study. As we adjusted for multiple testing in this study, these effects cannot be interpreted as significant but depict tendencies in the same direction as in our pilot, suggesting a decrease of problems in social responsiveness as observed by parents when there was a high AQR match rate. These tendencies might be useful for rethinking the role of parents as well as the selection of outcomes in future research.

In terms of clinical characteristics, the sample of the pilot and the present study included a high proportion of boys with a low cognitive level (IQ < 70). However, more children in this study have been assessed with ADOS module 1, meaning that more children had no or very limited verbal language skills. In addition, the sample scores of this study are significantly higher on the ADOS RRB domain. These results correspond with the higher amount of ratings on lower AQR modi (especially modus 1), which also reflect the occurrence of stereotyped behaviors and sensory issues in the present sample. Considering that children in this study were more affected by restricted and repetitive behavior than in the smaller pilot, changes might be hard to trace over a short time period, as RRB symptom severity as measured with the ADOS tends to persist or even worsen over time (Richler et al. 2010).

New to the present sample is that it included 30 participants from the UK. However, participants were recruited following the same study design and clinical effects in the UK were not clearly different from the other countries (Crawford et al. 2017). Chance may be the most suitable explanation.

Similarly to the pilot, it should be highlighted that the AQR match rate still significantly predicted autism severity in the sample included in the present study. Further, it should be noted that p-values in the previous study were not very low (all interaction terms p > 0.01; Mössler et al. 2019) and the present study still suggested some non-significant effects in the same direction.

### Limitations

The study’s limitations fall into two main categories: design and measures. Concerning the design, the observational design must be noted as a limitation of the present study as well as the previous study (Mössler et al. 2019). Only a randomized study could provide a definite answer to the question whether a successful attunement, as defined through the AQR match, leads to better outcomes, i.e. whether any correlation between this particular relational match and outcomes is causal.

Concerning the measures, both the predictors and the outcome measures are imperfect. First, the AQR was developed as a clinical tool for training and supervision (Schumacher et al. 2019) which would benefit from a cross-cultural validation. Used as a research tool, like we did for this study, transforming complex relational information on attunement processes to a simplified 0/1 match or mismatch rate might be too coarse. Furthermore, statistical analysis of the AQR data as applied in this study focused on match/mismatches created by the therapist. Hence, attunement appears more like a one-sided activity. However, following Tronicks (2008) research, all interaction partners involved contribute to the co-constitution of attunement processes. The same can be said about the IMT treatment fidelity ratings, where item 1 “musical and emotional attunement” only reflects the therapist’s working modes towards the child. Consequently, methodological as well as conceptual development regarding the co-constitution and assessment of attunement processes is needed.

Second, the relevance of autism severity in general, and ADOS scores in particular, as outcome measures has been disputed (Armstrong 2011; Gold and Bieleninik 2018; Turry 2018). Recent research has shown a remarkable stability of overall autism severity and autism symptoms, as measured by the ADOS, over time across childhood (Bieleninik et al. 2017a, b). It may be that music therapy has effects that are too subtle to be captured by the ADOS especially in the short-term. For future research, it might be important that the duration of the intervention, measurement time points as well as clinical outcomes better match the sample population. It might be that more specific outcomes directly related to the intervention should be used (Pijl et al. 2018; Vivanti et al. 2014). Furthermore, outcomes defined by and relevant to autistic people and their families should be focused in future studies (Broder-Fingert et al. 2017; Turry 2018).

Finally, we have to note that the gathered data might be too limited to reliably detect effects, since we substantially lack match ratings on lower AQR modi, especially modus 1 and 2 which were rated quite frequently for the child but not for the therapist and her/his interventions accordingly. That means that successful AQR attunement was mainly captured for (emerging) inter-personal aspects of communication and engagement (modi 3, 4, and 5), but not for those intra-personal and bodily aspects that might have been most relevant for the majority of the population presenting with no or very limited verbal language and with a high degree of RRB symptoms. Research has shown that changes in RRBs over time depend on the kind of RRB which has implications both for research (e.g. analysis of RRB data) and practice (Richler et al. 2010).

### Implications for Practice

RRB symptoms are more persistent or even increasing over time, interfere with all aspects of functioning in children with ASC including social and communication development in different ways (Richler et al. 2010), and they are less addressed by non-pharmacological treatments in general (Boyd et al. 2012). As we found that AQR match rate still significantly predicted autism severity in the present sample, meaning that a higher AQR match rate was significantly associated with a lower severity of ADOS symptoms at baseline, this study suggests, that attunement might be harder to achieve with children presenting with stronger symptoms. Even though the IMT guidelines for this study provided working modes on attunement, addressing the child’s sensory modalities, stereotype behavior and affective issues (Geretsegger et al. 2015), our findings indicate that issues related to RRB as well as affective dysregulation could be addressed to a greater extent by music therapists. Since music therapy offers unique potentials for addressing bodily expressions related to these symptom categories as for example stereotype repetition of movement, these findings might encourage music therapists to move and sound more in relation to children’s bodily expressions, creating opportunities for co-constituted experiences of attunement. In the improvised moment-to-moment interaction, distinctive for IMT as depicted in the opening case vignette, the expressive features of music, such as tempo, dynamics, timbre, and pitch, can correspond with the dynamic forms of affective, bodily-emotional expressions in the mutual, simultaneous or alternate interaction of child and therapist. IMT allows for a multisensory and dynamic interweaving of diverse modes of expression, movement, and interaction (Schmid 2017).

According to the AQR, music therapists in this study seem to be highly trained and qualified in interactive working modes (among others: musical games including mirroring or imitation, call-response activities) addressing the child’s exploration of her/his voice and music instruments, joined attention, social referencing, turn-taking, reciprocity, or a shared experience of joy all of which are important and meaningful when working with children with ASC. However, not all children might be able to already engage in *inter*active working modes, requiring a certain awareness of the interaction partner, as their *intra*personal needs might not sufficiently be addressed yet. According to the AQR it is assumed that children experiencing a disorganized sensory perception, restricted and repetitive interests (modus 0, 1) or affective arousals (modus 2) benefit from working modes focusing on the integration of senses and regulation of affects to found the basis for development of, for example, cross-modal perception, body coherence, and self-regulation—abilities known as basic intrapersonal elements in the emerging development of intersubjectivity (Meltzoff 1990; Stern 1985/2000; Trevarthen 1998). Following this theory, more attention should be given to the child’s bodily-affective regulation.

The possibility for the child and the therapist to move, having space and equipment that can support movement (e.g. trampoline, hammock) and sound making (including the possibility to be loud), as well as the possibility for the therapist to touch the child are important preconditions for being able to work with the child’s body. However, from our practical experiences from this study we know that these preconditions might not always be given in the particular setting in which music therapy is applied, especially when it is provided in school or kindergarten settings. We might assume that in settings were therapists have less possibilities to move with and touch a young child with ASC, it might be harder to attune to developmental needs related to the integration and regulation of senses and affects. Like in early infancy, these developmental achievements are based on movements, sounds and affects that are mutually attuned in timing, intensity and form between the interaction partners.

Further, since the main working focus was on the vocal and instrumental expression of the participating children, it can also be questioned whether IMT as applied in this study (Geretsegger et al. 2015) encouraged and equipped therapists sufficiently to use the child’s bodily expressions as musical means they can relate to. It might also be worth considering, whether it is easier to attune to a child’s vocal expression or music making on an instrument than to a bodily expression which might be hard to read. This might be a topic to be further discussed regarding the training and education of music therapists.

### Implications for Research

As mentioned above, our AQR based approach regarding the assessment of attunement presents with some limitations. It is thus important for future research to develop more accurate assessment procedures or tools that capture attunement as co-constituted processes. Additionally, training of the music therapists in the use of AQR or similar tools focusing on sensory and affective issues in autism might be important. It would be useful in the future to develop further studies in which “standard” IMT as, for example, applied in the TIME-A study is compared to AQR-informed music therapy.

Furthermore, it might be an advantage to focus on more homogeneous populations to be able to develop more specific treatment protocols and to choose more sensitive outcomes. The latter might especially be important in populations of children displaying with a low IQ, little or no verbal language, or a higher level of RRB symptoms. These children might be at risk to be underestimated when approaching them with a conventional conception of strengths and deficits instead of acknowledging a neurodiverse and individual variability of potentials (Courchesne et al. 2015). Relational changes connected to attunement processes might in a first place get visible on a bodily level, such as changes in movement, affective regulation and expression, or sensory perception and not on a generalized social skills level as measured in this study. Outcomes that are based on strength-informed approaches acknowledging intra-individual potentials are important to include in future studies (Courchesne et al. 2015). Moreover, no single tool will be able to capture changes meaningful to this diverse group of children. Since there is no existing consensus regarding reliable standardized outcome measures, a recent study recommends the use of multiple instruments (Provenzani et al. 2019). Variability and change over time can be related to several aspects of life such as functioning, community engagement, quality of life, specific child development domains, as well as context related outcomes (e.g. family, school settings) could be explored (Georgiades and Kasari 2018). A phenomenological long-term study on family-centered music therapy reports that mothers of children with ASC experienced enrichment in child and family quality of life, a strengthened understanding of the child’s resources and potentials, as well as improvements of social relationships within the family as important benefits from participating in music therapy (Thompson 2017). Compared to a limited psychological test setting, parents can draw on a broad spectrum of everyday life situations when evaluating their child. Hence, their evaluation might reflect the child’s capabilities and development to a more detailed level. Furthermore, they can report on their quality of life. Positive changes in this domain might even be more important to families than the reduction of single symptoms. Such perspectives of parents as well as autistic people on how they perceive and evaluate change need to be integrated when designing (music therapy) studies to get a more nuanced understanding about diverse development, individual improvements, and valuable changes meaningful to children with ASC and their families (McConachie et al. 2018). Furthermore, music therapy settings involving parents might increase opportunities for introducing change especially towards quality of life, parenting stress, and family relationships.

Final research questions concern how to investigate attunement as a relational factor within different cultures and without reducing it to a one-sided relational activity. Attunement might vary in its meaning and appearance in various cultures as involved in this study. Relational and bodily gestures (e.g. eye contact, touching) can have different meanings in its usage and it would therefore be interesting for future studies to look at cultural values that might impact attunement processes (Mössler et al. 2019). Furthermore, to capture attunement processes in research more comprehensively, including the child´s, therapist´s as well as assessor´s contributions to their emergence, calls for inclusive and embodied research methodologies as suggested by Halstead and colleagues (Halstead et al. 2017). Researchers themselves are getting involved in attunement processes while detecting and assessing them. They might therefore identify such processes through responses originating from their own bodies (Frank, 1995). Applying embodied practice in research, will contribute to a theoretical and methodological extension and expansion of research on attunement that acknowledges it as an intercorporeal phenomenon and activity (De Jaegher 2013).

As moving and musicking in music therapy have similarities to the musical interaction in early childhood (Malloch and Trevarthen 2009), moments of attunement and bodily synchronization both naturally occur and are created purposefully in music therapy. Findings of a recent pilot study conducting movement analysis suggest that music therapists are getting significantly more synchronized with children with ASC over the course of a therapy process (Dvir et al. 2019). Psychotherapy research has shown that movement synchrony is associated with positive affectivity and a positive quality of the relationship between the therapist and adult clients (Ramseyer and Tschacher 2011). Whether such synchronized moments of attunement in music therapy contribute to positive changes in children with ASC that are also traceable on a physiological basis might be a future field to explore. One promising pathway to follow might be further neurobiological examinations studying links between sensory, motor, affective, and social abilities through brain networks (Sharda et al. 2019). All these abilities can be addressed within IMT, which has already shown to improve functional brain connectivity (Sharda et al. 2018).

## Conclusion

This study does not replicate the positive results of the previous smaller study suggesting a significant association between musical and emotional qualities of attunement and symptom reduction in children with ASC (Mössler et al. 2019). We might see tendencies of improvement concerning the child’s social responsiveness as perceived by parents, which might provide valuable indications in terms of user involvement or the selections of outcomes when designing future studies. Our findings point to relational challenges when working with children with high levels of restricted and repetitive behavior, low IQ, and limited verbal language as attunement might be harder to achieve with this particular group. Consequently, they emphasize the importance of addressing bodily issues like sensory and movement deviations as well as affective dysregulation with greater awareness as already described by self-advocates (Robledo et al. 2012; Young 2011), parents (Amos 2013), music therapists (Berger 2002; Schumacher et al. 2019), and researchers from other fields (De Jaegher 2013; Trevarthen and Delafield-Butt 2013). As proposed within such theoretical thinking, we believe that music therapy can contribute to an integrated theory of autism that shifts the focus of attention on the human body, and its holistic role in cognition, emotion, and social interaction. Through their unique ability to dynamically and flexibly attune music to all kinds of expression, music therapists have the possibility to relate to particularities of moving, sounding, perceiving, and emoting, which define how children with ASC communicate, relate, and make sense of their world. Music therapy can be seen as resource-oriented approach connecting to the children’s strengths, interpreting particularities as possibilities and capabilities rather than deficits. In this sense, music therapy might also contribute to a neurodiversity view of autism, challenging the discussion of beneficial changes and outcomes and emphasizing the necessity of involving autistic people and their families in research activities to be able to create reliable findings that are valuable for them.

## Electronic supplementary material

Below is the link to the electronic supplementary material.

Supplementary file1 (DOCX 198 kb)
