# Supplementary material for: Attunement in Music Therapy for Young Children with Autism: Revisiting Qualities of Relationship as Mechanisms of Change
Source: J Autism Dev Disord. 2020 Mar 18;50(11):3921–34. doi: 10.1007/s10803-020-04448-w (PMC7560932; doi:10.1007/s10803-020-04448-w)
Supplement: Supplementary file 1 — Supplementary file1 (DOCX 198 kb) [file 10803_2020_4448_MOESM1_ESM.docx]

**Web Appendix**

**Appendix 1. Joint frequency distribution of the child’s and the therapist’s AQR modus by week**
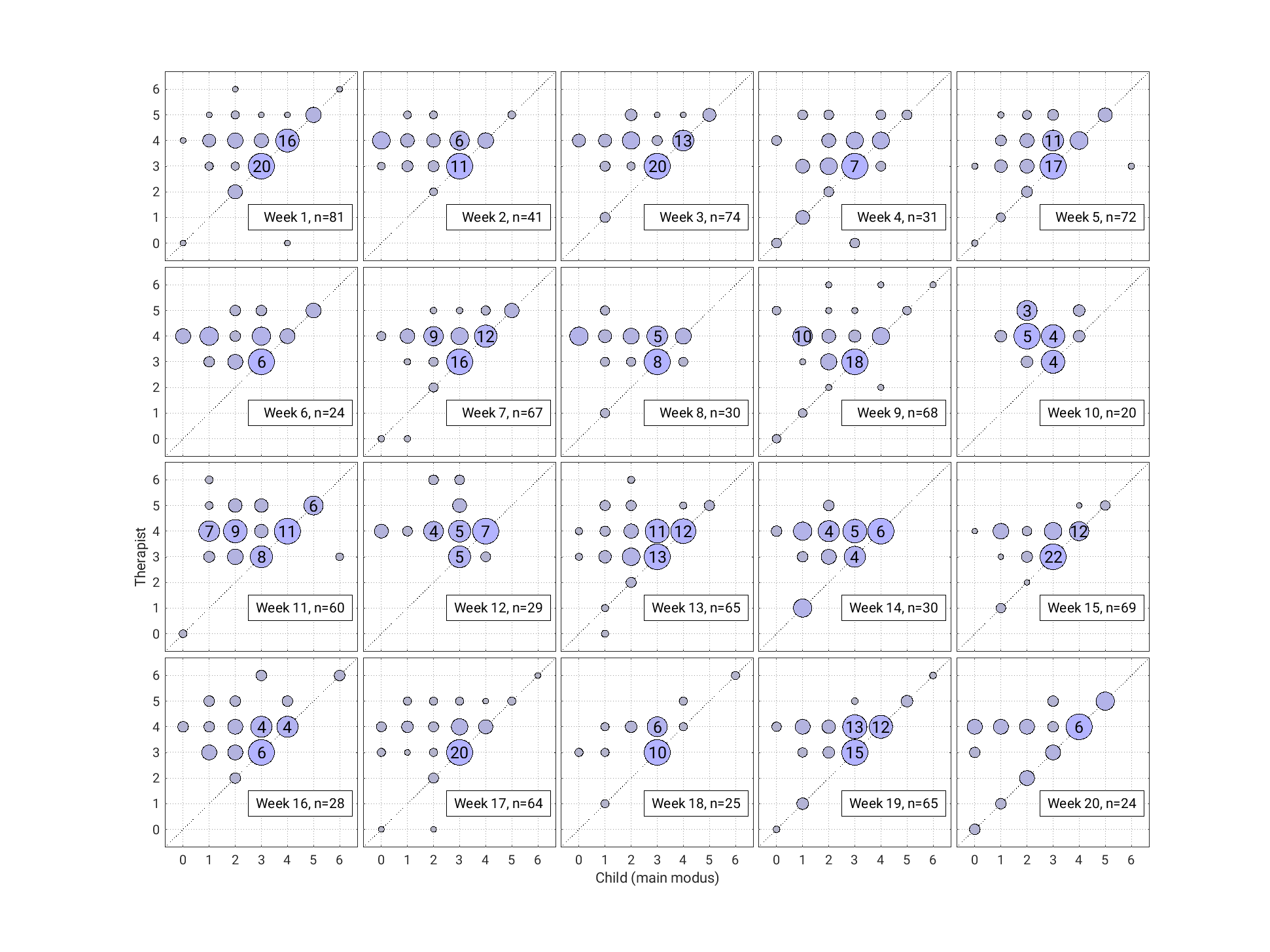


*Note*. Bubbles indicate frequency of occurrence of each combination of AQR modi for each week; the area of the bubbles is proportional to the number of sequences. For the highest frequencies, the number is shown within the bubble. Total number of sequences is shown in each plot.

**Appendix 2. All linear mixed effects models with AQR**

|  | Unadjusted Model | | | | |  | Model adjusted for site, diagnosis, MT intensity, therapist | | | | |
| --- | --- | --- | --- | --- | --- | --- | --- | --- | --- | --- | --- |
| ADOS Total | B | 95%CI | df | t | p-value |  | B | 95%CI | df | t | p-value |
| Intercept | 18.29 | (17.28, 19.30) | 194 | 35.29 | <.0001 |  | 15.68 | (10.99, 20.38) | 186 | 6.49 | <.0001 |
| AQR matchrate | -9.24 | (-13.30, -5.18) | 99 | -4.47 | <.0001 |  | -8.85 | (-12.97, -4.72) | 66 | -4.21 | 0.0001 |
| 5 months vs. BL | -0.8 | (-1.57, -0.04) | 194 | -2.05 | 0,0422 |  | -0.79 | (-1.57, -0.01) | 186 | -1.98 | 0.0494 |
| 12 months vs. BL | -1.84 | (-2.62, -1.07) | 194 | -4.64 | <.0001 |  | -1.93 | (-2.72, -1.14) | 186 | -4.75 | <.0001 |
| AQR match rate * (5 months vs. BL) | 0.44 | (-2.61, 3.49) | 194 | 0.28 | 0.7779 |  | 0.42 | (-1.53, 7.83) | 186 | 0.26 | 0.7932 |
| AQR match rate * (12 months vs. BL) | -0.62 | (-3.73, 2.48) | 194 | -0.39 | 0.6966 |  | -0.97 | (-4.91, 5.48) | 186 | -0.59 | 0.5551 |
|  |  |  |  |  |  |  |  |  |  |  |  |
|  | Unadjusted Model | | | | |  | Model adjusted for site, diagnosis, MT intensity, therapist | | | | |
| ADOS Social affect | B | 95%CI | df | t | p-value |  | B | 95%CI | df | t | p-value |
| Intercept | 14.38 | (13.53, 15.22) | 194 | 33.32 | <.0001 |  | 13.28 | (9.33, 17.23) | 186 | 6.53 | <.0001 |
| AQR matchrate | -7.21 | (-10.59, -3.83) | 99 | -4.19 | 0.0001 |  | -6.7 | (-10.14, -3.26) | 66 | -3.83 | 0.0003 |
| 5 months vs. BL | -0.75 | (-1.41, -0.09) | 194 | -2.22 | 0.0272 |  | -0.73 | (-1.40, -0.06) | 186 | -2.11 | 0.0365 |
| 12 months vs. BL | -1.63 | (-2.30, -0.96) | 194 | -4.74 | <.0001 |  | -1.66 | (-2.35, -0.98) | 186 | -4.72 | <.0001 |
| AQR match rate * (5 months vs. BL) | 0.4 | (-2.23, 3.03) | 194 | 0.3 | 0.7673 |  | 0.46 | (-2.18, 5.56) | 186 | 0.33 | 0.7397 |
| AQR match rate * (12 months vs. BL) | -0.36 | (-3.04, 2.32) | 194 | -0.26 | 0.7915 |  | -0.59 | (-4.65, 4.11) | 186 | -0.41 | 0.6795 |
|  |  |  |  |  |  |  |  |  |  |  |  |
|  | Unadjusted Model | | | | |  | Model adjusted for site, diagnosis, MT intensity ,therapist | | | | |
| ADOS Language and communication | B | 95%CI | df | t | p-value |  | B | 95%CI | df | t | p-value |
| Intercept | 3.46 | (3.16, 3.75) | 194 | 22.97 | <.0001 |  | 3.52 | (2.27, 4.78) | 186 | 5.44 | <.0001 |
| AQR matchrate | -1.36 | (-2.54, -0.18) | 99 | -2.27 | 0.0253 |  | -1.06 | (-2.24, 0.12) | 66 | -1.77 | 0.0811 |
| 5 months vs. BL | -0.29 | (-0.58, 0.00) | 194 | -1.94 | 0.0533 |  | -0.28 | (-0.57, 0.02) | 186 | -1.83 | 0.069 |
| 12 months vs. BL | -0.5 | (-0.79, -0.20) | 194 | -3.31 | 0.0011 |  | -0.49 | (-0.79, -0.19) | 186 | -3.17 | 0.0018 |
| AQR match rate * (5 months vs. BL) | 0.04 | (-1.11, 1.19) | 194 | 0.07 | 0.9408 |  | 0.04 | (-1.14, 1.27) | 186 | 0.06 | 0.9515 |
| AQR match rate * (12 months vs. BL) | -0.78 | (-1.95, 0.39) | 194 | -1.3 | 0.1954 |  | -0.79 | (-1.72, 1.04) | 186 | -1.28 | 0.2016 |
|  |  |  |  |  |  |  |  |  |  |  |  |
|  | Unadjusted Model | | | | |  | Model adjusted for site, diagnosis, MT intensity, therapist | | | | |
| ADOS Reciprocal social interaction | B | 95%CI | df | t | p-value |  | B | 95%CI | df | t | p-value |
| Intercept | 10.92 | (10.25, 11.60) | 194 | 31.57 | <.0001 |  | 9.86 | (6.71, 13.00) | 186 | 6.09 | <.0001 |
| AQR matchrate | -5.85 | (-8.56, -3.14) | 99 | -4.24 | 0.0001 |  | -5.66 | (-8.46, -2.86) | 66 | -3.97 | 0.0002 |
| 5 months vs. BL | -0.47 | (-1.02, 0.09) | 194 | -1.64 | 0.1031 |  | -0.45 | (-1.02, 0.12) | 186 | -1.55 | 0.1237 |
| 12 months vs. BL | -1.13 | (-1.70, -0.57) | 194 | -3.93 | 0.0001 |  | -1.18 | (-1.76, -0.60) | 186 | -3.97 | 0.0001 |
| AQR match rate * (5 months vs. BL) | 0.36 | (-1.86, 2.57) | 194 | 0.31 | 0.7538 |  | 0.43 | (-1.65, 4.57) | 186 | 0.36 | 0.7172 |
| **Appendix 2 (cont.)**  AQR match rate * (12 months vs. BL) | 0.41 | (-1.84, 2.67) | 194 | 0.36 | 0.7193 |  | 0.2 | (-3.48, 3.51) | 186 | 0.17 | 0.8654 |
|  |  |  |  |  |  |  |  |  |  |  |  |
|  | Unadjusted Model | | | | |  | Model adjusted for site, diagnosis, MT intensity, therapist | | | | |
| ADOS Restricted and repetitive behaviour | B | 95%CI | df | t | p-value |  | B | 95%CI | df | t | p-value |
| Intercept | 3.91 | (3.52, 4.30) | 194 | 19.75 | <.0001 |  | 2.18 | (0.49, 3.87) | 186 | 2.5 | 0.0133 |
| AQR matchrate | -2.03 | (-3.59, -0.48) | 99 | -2.58 | 0.0115 |  | -1.83 | (-3.42, -0.23) | 66 | -2.25 | 0.0279 |
| 5 months vs. BL | -0.05 | (-0.42, 0.32) | 194 | -0.26 | 0.794 |  | -0.06 | (-0.44, 0.31) | 186 | -0.32 | 0.7498 |
| 12 months vs. BL | -0.22 | (-0.60, 0.15) | 194 | -1.16 | 0.2472 |  | -0.28 | (-0.66, 0.11) | 186 | -1.4 | 0.1638 |
| AQR match rate * (5 months vs. BL) | 0.04 | (-1.43, 1.52) | 194 | 0.06 | 0.9555 |  | -0.04 | (0.05, 3.40) | 186 | -0.05 | 0.958 |
| AQR match rate * (12 months vs. BL) | -0.26 | (-1.76, 1.24) | 194 | -0.34 | 0.7331 |  | -0.39 | (-0.80, 2.94) | 186 | -0.5 | 0.6194 |
|  |  |  |  |  |  |  |  |  |  |  |  |
|  | Unadjusted Model | | | | |  | Model adjusted for site, diagnosis, MT intensity, therapist | | | | |
| Social responsiveness (SRS) total | B | 95%CI | df | t | p-value |  | B | 95%CI | df | t | p-value |
| Intercept | 96.07 | (90.38, 101.76) | 169 | 32.98 | <.0001 |  | 80.53 | (54.82, 106.25) | 162 | 6.08 | <.0001 |
| AQR matchrate | -13.49 | (-36.28, 9.31) | 99 | -1.16 | 0.2483 |  | -15.74 | (-38.14, 6.66) | 66 | -1.38 | 0.1725 |
| 5 months vs. BL | -6.6 | (-10.62, -2.58) | 169 | -3.2 | 0.0016 |  | -6.32 | (-10.44, -2.19) | 162 | -2.97 | 0.0034 |
| 12 months vs. BL | -8.62 | (-12.84, -4.41) | 169 | -3.99 | 0.0001 |  | -8.39 | (-12.75, -4.04) | 162 | -3.74 | 0.0003 |
| AQR match rate * (5 months vs. BL) | -16.06 | (-31.85, -0.27) | 169 | -1.99 | 0.0486 |  | -15.04 | (-11.81, 38.86) | 162 | -1.8 | 0.0737 |
| AQR match rate * (12 months vs. BL) | -3.24 | (-20.03, 13.56) | 169 | -0.38 | 0.7072 |  | -6.32 | (-25.70, 31.46) | 162 | -0.7 | 0.482 |
|  |  |  |  |  |  |  |  |  |  |  |  |
|  | Unadjusted Model | | | | |  | Model adjusted for site, diagnosis, MT intensity, therapist | | | | |
| SRS awareness | B | 95%CI | df | t | p-value |  | B | 95%CI | df | t | p-value |
| Intercept | 12.2 | (11.37, 13.03) | 171 | 28.68 | <.0001 |  | 9.84 | (6.00, 13.68) | 164 | 4,98 | <.0001 |
| AQR matchrate | -1.7 | (-5.03, 1.63) | 99 | -1 | 0.3185 |  | -1.1 | (-4.37, 2.16) | 66 | -0.66 | 0.5091 |
| 5 months vs. BL | -0.56 | (-1.22, 0.11) | 171 | -1.64 | 0.103 |  | -0.51 | (-1.19, 0.17) | 164 | -1.46 | 0.1467 |
| 12 months vs. BL | -0.31 | (-1.01, 0.38) | 171 | -0.88 | 0.3811 |  | -0.22 | (-0.94, 0.49) | 164 | -0.61 | 0.5441 |
| AQR match rate * (5 months vs. BL) | -1.76 | (-4.37, 0.85) | 171 | -1.32 | 0.1894 |  | -1.99 | (-1.65, 5.73) | 164 | -1.45 | 0.1492 |
| AQR match rate * (12 months vs. BL) | -0.39 | (-3.12, 2.33) | 171 | -0.28 | 0.7779 |  | -1.13 | (-3.09, 5.34) | 164 | -0.78 | 0.4366 |
|  |  |  |  |  |  |  |  |  |  |  |  |
|  | Unadjusted Model | | | | |  | Model adjusted for site, diagnosis, MT intensity, therapist | | | | |
| SRS cognition | B | 95%CI | df | t | p-value |  | B | 95%CI | df | t | p-value |
| Intercept | 18.21 | (17.03, 19.39) | 171 | 30.13 | <.0001 |  | 15.74 | (10.49, 20.98) | 164 | 5.82 | <.0001 |
| AQR matchrate | -3.28 | (-8.01, 1.45) | 99 | -1.36 | 0.1772 |  | -3.89 | (-8.61, 0.82) | 66 | -1.62 | 0.1101 |
| 5 months vs. BL | -1.13 | (-2.10, -0.15) | 171 | -2.26 | 0.0252 |  | -1.11 | (-2.09, -0.12) | 164 | -2.18 | 0.0304 |
| **Appendix 2 (cont.)**  12 months vs. BL | -1.37 | (-2.39, -0.35) | 171 | -2.63 | 0.0094 |  | -1.42 | (-2.46, -0.39) | 164 | -2.66 | 0.0085 |
| AQR match rate * (5 months vs. BL) | -3.92 | (-7.75, -0.08) | 171 | -1.99 | 0.0478 |  | -3.35 | (-3.09, 7.30) | 164 | -1.67 | 0.0961 |
| AQR match rate * (12 months vs. BL) | -0.37 | (-4.38, 3.64) | 171 | -0.18 | 0.8563 |  | -0.97 | (-5.48, 6.18) | 164 | -0.46 | 0.6463 |
|  |  |  |  |  |  |  |  |  |  |  |  |
|  | Unadjusted Model | | | | |  | Model adjusted for site, diagnosis, MT intensity ,therapist | | | | |
| SRS communication | B | 95%CI | df | t | p-value |  | B | 95%CI | df | t | p-value |
| Intercept | 32.1 | (29.98, 34.22) | 171 | 29.56 | <.0001 |  | 26.33 | (16.75, 35.91) | 164 | 5.34 | <.0001 |
| AQR matchrate | -3.97 | (-12.47, 4.53) | 99 | -0.92 | 0.3611 |  | -4.89 | (-13.43, 3.64) | 66 | -1.13 | 0.2646 |
| 5 months vs. BL | -2.64 | (-4.36, -0.92) | 171 | -3 | 0.0031 |  | -2.47 | (-4.24, -0.71) | 164 | -2.72 | 0.0073 |
| 12 months vs. BL | -2.83 | (-4.63, -1.03) | 171 | -3.07 | 0.0025 |  | -2.76 | (-4.63, -0.90) | 164 | -2.88 | 0.0046 |
| AQR match rate * (5 months vs. BL) | -3.97 | (-10.74, 2.79) | 171 | -1.15 | 0.2533 |  | -3.67 | (-4.64, 14.30) | 164 | -1.02 | 0.3079 |
| AQR match rate * (12 months vs. BL) | -2.12 | (-9.19, 4.96) | 171 | -0.58 | 0.56 |  | -2.78 | (-8.63, 12.65) | 164 | -0.73 | 0.4643 |
|  |  |  |  |  |  |  |  |  |  |  |  |
|  | Unadjusted Model | | | | |  | Model adjusted for site, diagnosis, MT intensity, therapist | | | | |
| SRS motivation | B | 95%CI | df | t | p-value |  | B | 95%CI | df | t | p-value |
| Intercept | 15.12 | (14.06, 16.17) | 171 | 28 | <.0001 |  | 14.21 | (9.61, 18.81) | 164 | 6 | <.0001 |
| AQR matchrate | -2.55 | (-6.78, 1.68) | 99 | -1.18 | 0,2394 |  | -3.05 | (-7.27, 1.17) | 66 | -1.42 | 0.1609 |
| 5 months vs. BL | -2.07 | (-2.98, -1.17) | 171 | -4.47 | <.0001 |  | -2.06 | (-2.99, -1.13) | 164 | -4.3 | <.0001 |
| 12 months vs. BL | -2.49 | (-3.44, -1.54) | 171 | -5.13 | <.0001 |  | -2.43 | (-3.41, -1.45) | 164 | -4.8 | <.0001 |
| AQR match rate * (5 months vs. BL) | -1.18 | (-4.75, 2.39) | 171 | -0.65 | 0.5189 |  | -1.05 | (-3.39, 5.68) | 164 | -0.56 | 0.5779 |
| AQR match rate * (12 months vs. BL) | 0.11 | (-3.63, 3.84) | 171 | 0.06 | 0.9555 |  | -0.27 | (-6.53, 3.68) | 164 | -0.13 | 0.8938 |
|  |  |  |  |  |  |  |  |  |  |  |  |
|  | Unadjusted Model | | | | |  | Model adjusted for site, diagnosis, MT intensity, therapist | | | | |
| SRS mannerisms | B | 95%CI | df | t | p-value |  | B | 95%CI | df | t | p-value |
| Intercept | 18.45 | (16.99, 19.91) | 171 | 24.67 | <.0001 |  | 13.84 | (7.06, 20.63) | 164 | 3.96 | 0.0001 |
| AQR matchrate | -1.99 | (-7.84, 3.86) | 99 | -0.67 | 0.5059 |  | -2.58 | (-8.49, 3.33) | 66 | -0.86 | 0.3955 |
| 5 months vs. BL | -1.01 | (-2.13, 0.11) | 171 | -1.76 | 0.0805 |  | -1 | (-2.15, 0.15) | 164 | -1.69 | 0.0929 |
| 12 months vs. BL | -1.71 | (-2.89, -0.54) | 171 | -2.84 | 0.005 |  | -1.62 | (-2.83, -0.41) | 164 | -2.6 | 0.0102 |
| AQR match rate * (5 months vs. BL) | -3.75 | (-8.16, 0.67) | 171 | -1.66 | 0.0996 |  | -3.48 | (-3.00, 10.39) | 164 | -1.49 | 0.1383 |
| AQR match rate * (12 months vs. BL) | -1.56 | (-6.18, 3.06) | 171 | -0.66 | 0.5113 |  | -2.3 | (-5.87, 9.20) | 164 | -0.93 | 0.3522 |
|  |  |  |  |  |  |  |  |  |  |  |  |

**Appendix 3. LMEs with IMT principles as predictors**

a) Total IMT score

|  | Unadjusted Model | | | | |  | Model adjusted for site, diagnosis, MT intensity, therapist | | | | |
| --- | --- | --- | --- | --- | --- | --- | --- | --- | --- | --- | --- |
| ADOS Total | B | 95%CI | df | t | p-value |  | B | 95%CI | df | t | p-value |
| Intercept | 17.52 | (16.20, 18.83) | 120 | 25.91 | <.0001 |  | 14.79 | (10.21, 19.37) | 112 | 6.23 | <.0001 |
| IMT Total | -0.18 | (-0.48, 0.13) | 61 | -1.16 | 0.2514 |  | -0.35 | (-0.66, -0.04) | 37 | -2.21 | 0.0333 |
| 5 months vs. BL | -0.14 | (-1.13, 0.84) | 120 | -0.28 | 0.7774 |  | -0.09 | (-1.10, 0.93) | 112 | -0.17 | 0.8675 |
| 12 months vs. BL | -0.68 | (-1.68, 0.31) | 120 | -1.34 | 0.182 |  | -0.74 | (-1.77, 0.29) | 112 | -1.39 | 0.1668 |
| IMT Total * (5 months vs. BL) | 0.01 | (-0.21, 0.24) | 120 | 0.09 | 0.9274 |  | 0.03 | (-0.47, 8.65) | 112 | 0.26 | 0.7961 |
| IMT Total * (12 months vs. BL) | -0.11 | (-0.33, 0.12) | 120 | -0.91 | 0.365 |  | -0.12 | (-5.80, 5.01) | 112 | -0.96 | 0.3394 |
|  |  |  |  |  |  |  |  |  |  |  |  |
|  | Unadjusted Model | | | | |  | Model adjusted for site, diagnosis, MT intensity, therapist | | | | |
| ADOS Social affect | B | 95%CI | df | t | p-value |  | B | 95%CI | df | t | p-value |
| Intercept | 14.02 | (12.94, 15.09) | 120 | 25.45 | <.0001 |  | 12.77 | (9.01, 16.53) | 112 | 6.56 | <.0001 |
| IMT Total | -0.18 | (-0.43, 0.06) | 61 | -1.45 | 0.1517 |  | -0.32 | (-0.58, -0.06) | 37 | -2.44 | 0.0196 |
| 5 months vs. BL | -0.3 | (-1.12, 0.52) | 120 | -0.72 | 0.4745 |  | -0.24 | (-1.09, 0.60) | 112 | -0.55 | 0.583 |
| 12 months vs. BL | -0.91 | (-1.73, -0.08) | 120 | -2.13 | 0.035 |  | -0.92 | (-1.77, -0.06) | 112 | -2.07 | 0.0411 |
| IMT Total * (5 months vs. BL) | 0.03 | (-0.16, 0.22) | 120 | 0.3 | 0.7623 |  | 0.04 | (-1.33, 6.16) | 112 | 0.39 | 0.6989 |
| IMT Total * (12 months vs. BL) | -0.02 | (-0.21, 0.17) | 120 | -0.19 | 0.851 |  | -0.04 | (-5.71, 3.14) | 112 | -0.35 | 0.7275 |
|  |  |  |  |  |  |  |  |  |  |  |  |
|  | Unadjusted Model | | | | |  | Model adjusted for site, diagnosis, MT intensity, therapist | | | | |
| ADOS Language and communication | B | 95%CI | df | t | p-value |  | B | 95%CI | df | t | p-value |
| Intercept | 3.35 | (3.00, 3.70) | 120 | 18.61 | <.0001 |  | 3,3 | (2.16, 4.43) | 112 | 5.6 | <.0001 |
| IMT Total | 0.02 | (-0.07, 0.10) | 61 | 0.38 | 0.7029 |  | -0.01 | (-0.10, 0.08) | 37 | -0.27 | 0.7861 |
| 5 months vs. BL | -0.38 | (-0.76, -0.01) | 120 | -1.98 | 0.0503 |  | -0.37 | (-0.76, 0.02) | 112 | -1.82 | 0.0709 |
| 12 months vs. BL | -0.39 | (-0.77, -0.01) | 120 | -2.02 | 0.0458 |  | -0.37 | (-0.76, 0.03) | 112 | -1.79 | 0.0761 |
| IMT Total * (5 months vs. BL) | -0.03 | (-0.12, 0.05) | 120 | -0.73 | 0.4674 |  | -0.03 | (-0.78, 1.46) | 112 | -0.68 | 0.5003 |
| IMT Total * (12 months vs. BL) | 0 | (-0.09, 0.08) | 120 | -0.1 | 0.9203 |  | -0.02 | (-1.74, 0.90) | 112 | -0.36 | 0.723 |
|  |  |  |  |  |  |  |  |  |  |  |  |
|  | Unadjusted Model | | | | |  | Model adjusted for site, diagnosis, MT intensity, therapist | | | | |
| ADOS Reciprocal social interaction | B | 95%CI | df | t | p-value |  | B | 95%CI | df | t | p-value |
| Intercept | 10.67 | (9.82, 11.51) | 120 | 24.6 | <.0001 |  | 9.47 | (6.52, 12.42) | 112 | 6.2 | <.0001 |
| IMT Total | -0.2 | (-0.39, -0.00) | 61 | -2 | 0,0496 |  | -0.31 | (-0.51, -0.10) | 37 | -2.97 | 0.0052 |
| 5 months vs. BL | 0.08 | (-0.58, 0.74) | 120 | 0.23 | 0,8154 |  | 0.13 | (-0.56, 0.81) | 112 | 0.36 | 0.7175 |
| **Appendix 3a (cont.)**  12 months vs. BL | -0.52 | (-1.19, 0.15) | 120 | -1.52 | 0.1305 |  | -0.56 | (-1.25, 0.13) | 112 | -1.56 | 0.1227 |
| IMT Total * (5 months vs. BL) | 0.06 | (-0.09, 0.21) | 120 | 0.79 | 0.4313 |  | 0.07 | (-0.85, 5.01) | 112 | 0.87 | 0.3887 |
| IMT Total * (12 months vs. BL) | -0.01 | (-0.17, 0.14) | 120 | -0.18 | 0.8594 |  | -0.02 | (-4.31, 2.62) | 112 | -0.23 | 0.8198 |
|  |  |  |  |  |  |  |  |  |  |  |  |
|  | Unadjusted Model | | | | |  | Model adjusted for site, diagnosis, MT intensity, therapist | | | | |
| ADOS Restricted and repetitive behaviour | B | 95%CI | df | t | p-value |  | B | 95%CI | df | t | p-value |
| Intercept | 3.5 | (2.98, 4.02) | 120 | 13.21 | <.0001 |  | 2.15 | (0.29, 4.01) | 112 | 2.23 | 0.028 |
| IMT Total | 0 | (-0.12, 0.12) | 61 | 0.06 | 0.9495 |  | -0.06 | (-0.20, 0.07) | 37 | -0.95 | 0.3488 |
| 5 months vs. BL | 0.16 | (-0.32, 0.64) | 120 | 0.65 | 0.5198 |  | 0.15 | (-0.34, 0.65) | 112 | 0.59 | 0.5533 |
| 12 months vs. BL | 0.22 | (-0.27, 0.70) | 120 | 0.88 | 0.3815 |  | 0.18 | (-0.33, 0.68) | 112 | 0.67 | 0.5015 |
| IMT Total * (5 months vs. BL) | -0.02 | (-0.13, 0.09) | 120 | -0.33 | 0.7414 |  | -0.01 | (-0.41, 3.10) | 112 | -0.13 | 0.8959 |
| IMT Total * (12 months vs. BL) | -0.09 | (-0.20, 0.02) | 120 | -1.53 | 0.1283 |  | -0.08 | (-1.12, 2.99) | 112 | -1.35 | 0.1793 |
|  |  |  |  |  |  |  |  |  |  |  |  |
|  | Unadjusted Model | | | | |  | Model adjusted for site, diagnosis, MT intensity, therapist | | | | |
| Social responsiveness (SRS) total | B | 95%CI | df | t | p-value |  | B | 95%CI | df | t | p-value |
| Intercept | 94.4 | (87.23, 101.56) | 110 | 25.66 | <.0001 |  | 80.86 | (54.98, 106.74) | 103 | 6.03 | <.0001 |
| IMT Total | -0.74 | (-2.39, 0.92) | 61 | -0.88 | 0.3847 |  | -1.53 | (-3.35, 0.29) | 37 | -1.66 | 0.1055 |
| 5 months vs. BL | -6.74 | (-11.50, -1.99) | 110 | -2.76 | 0.0067 |  | -6.08 | (-11.01, -1.16) | 103 | -2.38 | 0.0189 |
| 12 months vs. BL | -10.35 | (-15.36, -5.34) | 110 | -4.02 | 0.0001 |  | -10.63 | (-15.87, -5.39) | 103 | -3.92 | 0.0002 |
| IMT Total * (5 months vs. BL) | -0.49 | (-1.57, 0.59) | 110 | -0.88 | 0.38 |  | -0.49 | (-11.91, 39.67) | 103 | -0.82 | 0.4122 |
| IMT Total * (12 months vs. BL) | 0.35 | (-0.74, 1.44) | 110 | 0.63 | 0.5332 |  | 0.28 | (-39.45, 21.43) | 103 | 0.46 | 0.6461 |
|  |  |  |  |  |  |  |  |  |  |  |  |
|  | Unadjusted Model | | | | |  | Model adjusted for site, diagnosis, MT intensity, therapist | | | | |
| SRS awareness | B | 95%CI | df | t | p-value |  | B | 95%CI | df | t | p-value |
| Intercept | 12 | (10.98, 13.02) | 111 | 22.94 | <.0001 |  | 9.39 | (5.77, 13.01) | 104 | 5 | <.0001 |
| IMT Total | -0.11 | (-0.35, 0.12) | 61 | -0.94 | 0.3525 |  | -0.23 | (-0.48, 0.02) | 37 | -1.83 | 0.0747 |
| 5 months vs. BL | -0.77 | (-1.56, 0.03) | 111 | -1.88 | 0.0623 |  | -0.71 | (-1.53, 0.11) | 104 | -1.66 | 0.099 |
| 12 months vs. BL | -0.71 | (-1.55, 0.13) | 111 | -1.65 | 0.1017 |  | -0.72 | (-1.60, 0.16) | 104 | -1.59 | 0.1157 |
| IMT Total * (5 months vs. BL) | -0.03 | (-0.21, 0.15) | 111 | -0.29 | 0.7748 |  | -0.03 | (-1.10, 6.10) | 104 | -0.3 | 0.7656 |
| IMT Total * (12 months vs. BL) | 0.14 | (-0.04, 0.33) | 111 | 1.53 | 0.1294 |  | 0.15 | (-4.62, 3.92) | 104 | 1.47 | 0.1458 |
|  |  |  |  |  |  |  |  |  |  |  |  |
|  | Unadjusted Model | | | | |  | Model adjusted for site, diagnosis, MT intensity, therapist | | | | |
| SRS cognition | B | 95%CI | df | t | p-value |  | B | 95%CI | df | t | p-value |
| **Appendix 3a (cont.)**  Intercept | 17.67 | (16.15, 19.19) | 111 | 22.62 | <.0001 |  | 16.56 | (11.14, 21.99) | 104 | 5.89 | <.0001 |
| IMT Total | -0.24 | (-0.59, 0.11) | 61 | -1.32 | 0.1903 |  | -0.42 | (-0.81, -0.04) | 37 | -2.17 | 0.0367 |
| 5 months vs. BL | -1.38 | (-2.62, -0.15) | 111 | -2.18 | 0.0317 |  | -1.32 | (-2.57, -0.08) | 104 | -2.05 | 0.0432 |
| 12 months vs. BL | -1.87 | (-3.17, -0.56) | 111 | -2.78 | 0.0065 |  | -2.11 | (-3.44, -0.77) | 104 | -3.05 | 0.0029 |
| IMT Total * (5 months vs. BL) | -0.04 | (-0.32, 0.24) | 111 | -0.26 | 0.7922 |  | 0.03 | (-3.81, 6.90) | 104 | 0.18 | 0.8569 |
| IMT Total * (12 months vs. BL) | 0.2 | (-0.09, 0.48) | 111 | 1.35 | 0.1805 |  | 0.24 | (-9.26, 3.31) | 104 | 1.57 | 0.1197 |
|  |  |  |  |  |  |  |  |  |  |  |  |
|  | Unadjusted Model | | | | |  | Model adjusted for site, diagnosis, MT intensity, therapist | | | | |
| SRS communication | B | 95%CI | df | t | p-value |  | B | 95%CI | df | t | p-value |
| Intercept | 32.4 | (29.67, 35.13) | 111 | 23.1 | <.0001 |  | 27.4 | (17.66, 37.14) | 104 | 5.43 | <.0001 |
| IMT Total | -0.34 | (-0.97, 0.29) | 61 | -1.06 | 0.295 |  | -0.6 | (-1.27, 0.06) | 37 | -1.79 | 0.0814 |
| 5 months vs. BL | -3.15 | (-5.27, -1.02) | 111 | -2.89 | 0.0046 |  | -2.85 | (-5.06, -0.63) | 104 | -2.48 | 0.0148 |
| 12 months vs. BL | -3.88 | (-6.12, -1.63) | 111 | -3.36 | 0.0011 |  | -4 | (-6.37, -1.64) | 104 | -3.26 | 0.0015 |
| IMT Total * (5 months vs. BL) | -0.12 | (-0.60, 0.37) | 111 | -0.46 | 0.6445 |  | -0.18 | (-4.47, 14.91) | 104 | -0.69 | 0.4924 |
| IMT Total * (12 months vs. BL) | 0.1 | (-0.39, 0.59) | 111 | 0.39 | 0.6988 |  | 0.03 | (-13.81, 9.17) | 104 | 0.1 | 0.9241 |
|  |  |  |  |  |  |  |  |  |  |  |  |
|  | Unadjusted Model | | | | |  | Model adjusted for site, diagnosis, MT intensity, therapist | | | | |
| SRS motivation | B | 95%CI | df | t | p-value |  | B | 95%CI | df | t | p-value |
| Intercept | 14.54 | (13.29, 15.79) | 111 | 22.7 | <.0001 |  | 14.16 | (9.76, 18.55) | 104 | 6.21 | <.0001 |
| IMT Total | -0.1 | (-0.39, 0.19) | 61 | -0.68 | 0.4963 |  | -0.23 | (-0.54, 0.08) | 37 | -1.47 | 0.1488 |
| 5 months vs. BL | -1.94 | (-3.00, -0.89) | 111 | -3.59 | 0.0005 |  | -1,88 | (-2.98, -0.79) | 104 | -3.32 | 0.0012 |
| 12 months vs. BL | -1.93 | (-3.04, -0.81) | 111 | -3.37 | 0.001 |  | -1.88 | (-3.05, -0.71) | 104 | -3.11 | 0.0024 |
| IMT Total * (5 months vs. BL) | -0.14 | (-0.38, 0.10) | 111 | -1.13 | 0,2602 |  | -0.15 | (-3.61, 5.13) | 104 | -1.1 | 0.2735 |
| IMT Total * (12 months vs. BL) | -0.02 | (-0.27, 0.22) | 111 | -0.18 | 0,8589 |  | -0.05 | (-8.58, 1.76) | 104 | -0.39 | 0.7001 |
|  |  |  |  |  |  |  |  |  |  |  |  |
|  | Unadjusted Model | | | | |  | Model adjusted for site, diagnosis, MT intensity, therapist | | | | |
| SRS mannerisms | B | 95%CI | df | t | p-value |  | B | 95%CI | df | t | p-value |
| Intercept | 17.79 | (15.96, 19.63) | 111 | 18.88 | <.0001 |  | 12.81 | (5.99, 19.63) | 104 | 3.62 | 0.0004 |
| IMT Total | 0.05 | (-0.37, 0.47) | 61 | 0.24 | 0.813 |  | 0.01 | (-0.47, 0.48) | 37 | 0.03 | 0.9735 |
| 5 months vs. BL | -0.69 | (-2.07, 0.69) | 111 | -0.97 | 0.3332 |  | -0.6 | (-2.03, 0.83) | 104 | -0.81 | 0.4182 |
| 12 months vs. BL | -2.12 | (-3.58, -0.66) | 111 | -2.83 | 0.0055 |  | -2.09 | (-3.62, -0.56) | 104 | -2.64 | 0.0097 |
| IMT Total * (5 months vs. BL) | -0.11 | (-0.43, 0.20) | 111 | -0.7 | 0.4856 |  | -0.08 | (-2.42, 11.16) | 104 | -0.47 | 0.6395 |
| IMT Total * (12 months vs. BL) | -0.06 | (-0.37, 0.26) | 111 | -0.34 | 0.7316 |  | -0.07 | (-7.73, 8.33) | 104 | -0.42 | 0.6788 |

**Appendix 3. LMEs with IMT principles as predictors**

b) Score of IMT principle 1 (musical and emotional attunement)

|  | Unadjusted Model | | | | |  | Model adjusted for site, diagnosis, MT intensity, therapist | | | | |
| --- | --- | --- | --- | --- | --- | --- | --- | --- | --- | --- | --- |
| ADOS Total | B | 95%CI | df | t | p-value |  | B | 95%CI | df | t | p-value |
| Intercept | 17.52 | (16.19, 18.84) | 120 | 25.7 | <.0001 |  | 14.58 | (9.91, 19.25) | 112 | 6.02 | <.0001 |
| IMT P1 | -0.84 | (-3.35, 1.67) | 61 | -0.66 | 0.5126 |  | -2.12 | (-4.67, 0.43) | 37 | -1.64 | 0.1099 |
| 5 months vs. BL | -0.14 | (-1.13, 0.84) | 120 | -0.28 | 0.7778 |  | -0.08 | (-1.10, 0.93) | 112 | -0.16 | 0.8729 |
| 12 months vs. BL | -0.68 | (-1.68, 0.31) | 120 | -1.34 | 0.1842 |  | -0.74 | (-1.77, 0.29) | 112 | -1.39 | 0.1668 |
| IMT P1 * (5 months vs. BL) | -0.22 | (-2.06, 1.62) | 120 | -0.23 | 0.8164 |  | -0.05 | (-0.30, 8.99) | 112 | -0.05 | 0.9583 |
| IMT P1 * (12 months vs. BL) | -0.81 | (-2.66, 1.05) | 120 | -0.85 | 0.399 |  | -0.93 | (-5.23, 5.71) | 112 | -0.93 | 0.3542 |
|  |  |  |  |  |  |  |  |  |  |  |  |
|  | Unadjusted Model | | | | |  | Model adjusted for site, diagnosis, MT intensity, therapist | | | | |
| ADOS Social affect | B | 95%CI | df | t | p-value |  | B | 95%CI | df | t | p-value |
| Intercept | 14.02 | (12.93, 15.10) | 120 | 25.24 | <.0001 |  | 12.56 | (8.72, 16.40) | 112 | 6.31 | <.0001 |
| IMT P1 | -0.99 | (-3.03, 1.06) | 61 | -0.95 | 0.3468 |  | -1.97 | (-4.10, 0.15) | 37 | -1.84 | 0.0745 |
| 5 months vs. BL | -0.3 | (-1.12, 0.52) | 120 | -0.72 | 0.4748 |  | -0.24 | (-1.08, 0.61) | 112 | -0.54 | 0.5887 |
| 12 months vs. BL | -0.91 | (-1.73, -0.08) | 120 | -2.13 | 0.0354 |  | -0.92 | (-1.77, -0.06) | 112 | -2.07 | 0.0412 |
| IMT P1 * (5 months vs. BL) | -0.04 | (-1.57, 1.50) | 120 | -0.05 | 0.9624 |  | 0.07 | (-1.13, 6.51) | 112 | 0.08 | 0.9328 |
| IMT P1 * (12 months vs. BL) | -0.17 | (-1.72, 1.37) | 120 | -0.22 | 0.8283 |  | -0.31 | (-5.17, 3.78) | 112 | -0.37 | 0.7113 |
|  |  |  |  |  |  |  |  |  |  |  |  |
|  | Unadjusted Model | | | | |  | Model adjusted for site, diagnosis, MT intensity, therapist | | | | |
| ADOS Language and communication | B | 95%CI | df | t | p-value |  | B | 95%CI | df | t | p-value |
| Intercept | 3.35 | (3.00, 3.70) | 120 | 18.64 | <.0001 |  | 3.29 | (2.15, 4.42) | 112 | 5.57 | <.0001 |
| IMT P1 | 0.21 | (-0.46, 0.87) | 61 | 0.61 | 0.5434 |  | -0.03 | (-0.74, 0.67) | 37 | -0.09 | 0.9272 |
| 5 months vs. BL | -0.38 | (-0.76, -0.01) | 120 | -1.98 | 0.0497 |  | -0.37 | (-0.76, 0.02) | 112 | -1.83 | 0.07 |
| 12 months vs. BL | -0.39 | (-0.77, -0.01) | 120 | -2.02 | 0.0452 |  | -0.37 | (-0.76, 0.03) | 112 | -1.8 | 0.075 |
| IMT P1 * (5 months vs. BL) | -0.39 | (-1.09, 0.31) | 120 | -1.09 | 0.2768 |  | -0.37 | (-0.76, 1.48) | 112 | -0.98 | 0.3308 |
| IMT P1 * (12 months vs. BL) | -0.06 | (-0.77, 0.64) | 120 | -0.17 | 0.863 |  | -0.14 | (-1.69, 0.93) | 112 | -0.38 | 0.7082 |
|  |  |  |  |  |  |  |  |  |  |  |  |
|  | Unadjusted Model | | | | |  | Model adjusted for site, diagnosis, MT intensity, therapist | | | | |
| ADOS Reciprocal social interaction | B | 95%CI | df | t | p-value |  | B | 95%CI | df | t | p-value |
| Intercept | 10.67 | (9.81, 11.52) | 120 | 24.29 | <.0001 |  | 9.27 | (6.24, 12.31) | 112 | 5.89 | <.0001 |
| IMT P1 | -1.19 | (-2.81, 0.43) | 61 | -1.45 | 0.1526 |  | -1.93 | (-3.62, -0.25) | 37 | -2.26 | 0.0297 |
| 5 months vs. BL | 0.08 | (-0.58, 0.74) | 120 | 0.23 | 0.8158 |  | 0.13 | (-0.55, 0.82) | 112 | 0.37 | 0.7105 |
| **Appendix 3b (cont.)**  12 months vs. BL | -0.52 | (-1.19, 0.15) | 120 | -1.52 | 0.1322 |  | -0.56 | (-1.25, 0.14) | 112 | -1.55 | 0.1234 |
| IMT P1 * (5 months vs. BL) | 0.36 | (-0.88, 1.59) | 120 | 0.56 | 0.5772 |  | 0.44 | (-0.68, 5.35) | 112 | 0.66 | 0.5104 |
| IMT P1 * (12 months vs. BL) | -0.11 | (-1.36, 1.14) | 120 | -0.17 | 0.8646 |  | -0.16 | (-3.85, 3.22) | 112 | -0.24 | 0.8088 |
|  |  |  |  |  |  |  |  |  |  |  |  |
|  | Unadjusted Model | | | | |  | Model adjusted for site, diagnosis, MT intensity, therapist | | | | |
| ADOS Restricted and repetitive behaviour | B | 95%CI | df | t | p-value |  | B | 95%CI | df | t | p-value |
| Intercept | 3.5 | (2.98, 4.02) | 120 | 13.18 | <.0001 |  | 2.12 | (0.25, 3.99) | 112 | 2.19 | 0.0307 |
| IMT P1 | 0.15 | (-0.83, 1.12) | 61 | 0.29 | 0.7713 |  | -0.26 | (-1.34, 0.82) | 37 | -0.47 | 0.639 |
| 5 months vs. BL | 0.16 | (-0.32, 0.64) | 120 | 0.64 | 0.5209 |  | 0.15 | (-0.35, 0.65) | 112 | 0.59 | 0.5537 |
| 12 months vs. BL | 0.22 | (-0.27, 0.70) | 120 | 0.88 | 0.3807 |  | 0.17 | (-0.33, 0.68) | 112 | 0.66 | 0.5108 |
| IMT P1 * (5 months vs. BL) | -0.18 | (-1.08, 0.72) | 120 | -0.4 | 0.6926 |  | -0.12 | (-0.36, 3.20) | 112 | -0.25 | 0.8027 |
| IMT P1 * (12 months vs. BL) | -0.63 | (-1.53, 0.28) | 120 | -1.35 | 0.1795 |  | -0.61 | (-1.03, 3.13) | 112 | -1.25 | 0.2136 |
|  |  |  |  |  |  |  |  |  |  |  |  |
|  | Unadjusted Model | | | | |  | Model adjusted for site, diagnosis, MT intensity, therapist | | | | |
| Social responsiveness (SRS) total | B | 95%CI | df | t | p-value |  | B | 95%CI | df | t | p-value |
| Intercept | 94.4 | (87.23, 101.57) | 110 | 25.64 | <.0001 |  | 80.17 | (54.22, 106.13) | 103 | 5.96 | <.0001 |
| IMT P1 | -6.89 | (-20.43, 6.65) | 61 | -1 | 0.3211 |  | -12.68 | (-27.26, 1.90) | 37 | -1.71 | 0.095 |
| 5 months vs. BL | -6.72 | (-11.48, -1.96) | 110 | -2.75 | 0.0069 |  | -6.1 | (-11.02, -1.17) | 103 | -2.39 | 0.0188 |
| 12 months vs. BL | -10.37 | (-15.38, -5.35) | 110 | -4.03 | 0.0001 |  | -10.65 | (-15.89, -5.41) | 103 | -3.92 | 0.0002 |
| IMT P1 * (5 months vs. BL) | -1.87 | (-10.75, 7.01) | 110 | -0.41 | 0.6827 |  | -1.63 | (-10.98, 40.69) | 103 | -0.34 | 0.7355 |
| IMT P1 * (12 months vs. BL) | 4.66 | (-4.26, 13.58) | 110 | 1.02 | 0.3114 |  | 4.25 | (-37.58, 22.99) | 103 | 0.87 | 0.384 |
|  |  |  |  |  |  |  |  |  |  |  |  |
|  | Unadjusted Model | | | | |  | Model adjusted for site, diagnosis, MT intensity, therapist | | | | |
| SRS awareness | B | 95%CI | df | t | p-value |  | B | 95%CI | df | t | p-value |
| Intercept | 12 | (10.98, 13.02) | 111 | 22.91 | <.0001 |  | 9.31 | (5.67, 12.94) | 104 | 4.94 | <.0001 |
| IMT P1 | -0.89 | (-2.82, 1.04) | 61 | -0.91 | 0.3675 |  | -1.75 | (-3.74, 0.23) | 37 | -1.74 | 0.0904 |
| 5 months vs. BL | -0.77 | (-1.56, 0.03) | 111 | -1.88 | 0.063 |  | -0.71 | (-1.53, 0.11) | 104 | -1.67 | 0.0985 |
| 12 months vs. BL | -0.71 | (-1.55, 0.13) | 111 | -1.65 | 0.1008 |  | -0.72 | (-1.60, 0.16) | 104 | -1.58 | 0.1179 |
| IMT P1 * (5 months vs. BL) | -0.05 | (-1.54, 1.43) | 111 | -0.07 | 0.9442 |  | -0.05 | (-1.00, 6.22) | 104 | -0.06 | 0.9531 |
| IMT P1 * (12 months vs. BL) | 1.18 | (-0.32, 2.68) | 111 | 1.53 | 0.1282 |  | 1.21 | (-4.36, 4.13) | 104 | 1.48 | 0.141 |
|  |  |  |  |  |  |  |  |  |  |  |  |
|  | Unadjusted Model | | | | |  | Model adjusted for site, diagnosis, MT intensity, therapist | | | | |
| SRS cognition | B | 95%CI | df | t | p-value |  | B | 95%CI | df | t | p-value |
| **Appendix 3b (cont.)**  Intercept | 17.67 | (16.14, 19.19) | 111 | 22.6 | <.0001 |  | 16.44 | (10.98, 21.90) | 104 | 5.81 | <.0001 |
| IMT P1 | -2.15 | (-5.03, 0.72) | 61 | -1.47 | 0.1463 |  | -3.38 | (-6.43, -0.32) | 37 | -2.18 | 0.036 |
| 5 months vs. BL | -1.38 | (-2.62, -0.14) | 111 | -2.17 | 0.0321 |  | -1.33 | (-2.58, -0.08) | 104 | -2.05 | 0.0424 |
| 12 months vs. BL | -1.87 | (-3.18, -0.56) | 111 | -2.78 | 0.0064 |  | -2.1 | (-3.44, -0.77) | 104 | -3.04 | 0.003 |
| IMT P1 * (5 months vs. BL) | 0.3 | (-2.02, 2.61) | 111 | 0.25 | 0.8038 |  | 0.86 | (-3.64, 7.12) | 104 | 0.71 | 0.4816 |
| IMT P1 * (12 months vs. BL) | 1.87 | (-0.47, 4.20) | 111 | 1.56 | 0.1225 |  | 2.18 | (-8.89, 3.67) | 104 | 1.76 | 0.0815 |
|  |  |  |  |  |  |  |  |  |  |  |  |
|  | Unadjusted Model | | | | |  | Model adjusted for site, diagnosis, MT intensity, therapist | | | | |
| SRS communication | B | 95%CI | df | t | p-value |  | B | 95%CI | df | t | p-value |
| Intercept | 32.4 | (29.66, 35.14) | 111 | 23.03 | <.0001 |  | 27.14 | (17.34, 36.94) | 104 | 5.34 | <.0001 |
| IMT P1 | -2.94 | (-8.11, 2.24) | 61 | -1.12 | 0.2689 |  | -4.95 | (-10.32, 0.41) | 37 | -1.82 | 0.077 |
| 5 months vs. BL | -3.14 | (-5.26, -1.01) | 111 | -2.88 | 0.0048 |  | -2.86 | (-5.09, -0.64) | 104 | -2.49 | 0.0145 |
| 12 months vs. BL | -3.88 | (-6.13, -1.63) | 111 | -3.36 | 0.0011 |  | -4.01 | (-6.38, -1.63) | 104 | -3.26 | 0.0015 |
| IMT P1 * (5 months vs. BL) | 0.34 | (-3.63, 4.32) | 111 | 0.17 | 0.8669 |  | -0.07 | (-4.17, 15.31) | 104 | -0.03 | 0.9729 |
| IMT P1 * (12 months vs. BL) | 1.23 | (-2.77, 5.24) | 111 | 0.6 | 0.55 |  | 0.72 | (-13.00, 9.93) | 104 | 0.33 | 0.7436 |
|  |  |  |  |  |  |  |  |  |  |  |  |
|  | Unadjusted Model | | | | |  | Model adjusted for site, diagnosis, MT intensity, therapist | | | | |
| SRS motivation | B | 95%CI | df | t | p-value |  | B | 95%CI | df | t | p-value |
| Intercept | 14.54 | (13.29, 15.79) | 111 | 22.69 | <.0001 |  | 14.07 | (9.68, 18.47) | 104 | 6.18 | <.0001 |
| IMT P1 | -1.2 | (-3.56, 1.15) | 61 | -1 | 0.32 |  | -2.22 | (-4.71, 0.27) | 37 | -1.75 | 0.0877 |
| 5 months vs. BL | -1.94 | (-3.00, -0.88) | 111 | -3.58 | 0.0005 |  | -1.89 | (-2.99, -0.79) | 104 | -3.32 | 0.0012 |
| 12 months vs. BL | -1.93 | (-3.05, -0.81) | 111 | -3.36 | 0.0011 |  | -1.89 | (-3.07, -0.72) | 104 | -3.11 | 0.0024 |
| IMT P1 * (5 months vs. BL) | -0.54 | (-2.52, 1.44) | 111 | -0.53 | 0.5968 |  | -0.57 | (-3.46, 5.26) | 104 | -0.53 | 0.5986 |
| IMT P1 * (12 months vs. BL) | 0.32 | (-1.67, 2.32) | 111 | 0.32 | 0.7521 |  | 0.13 | (-8.30, 1.95) | 104 | 0.12 | 0.9035 |
|  |  |  |  |  |  |  |  |  |  |  |  |
|  | Unadjusted Model | | | | |  | Model adjusted for site, diagnosis, MT intensity, therapist | | | | |
| SRS mannerisms | B | 95%CI | df | t | p-value |  | B | 95%CI | df | t | p-value |
| Intercept | 17.79 | (15.96, 19.63) | 111 | 18.88 | <.0001 |  | 12.72 | (5.92, 19.52) | 104 | 3.61 | 0.0005 |
| IMT P1 | 0.29 | (-3.18, 3.76) | 61 | 0.16 | 0.8698 |  | 0.03 | (-3.78, 3.85) | 37 | 0.02 | 0.9859 |
| 5 months vs. BL | -0.69 | (-2.07, 0.70) | 111 | -0.97 | 0.3355 |  | -0.61 | (-2.04, 0.82) | 104 | -0.82 | 0.4127 |
| 12 months vs. BL | -2.12 | (-3.58, -0.66) | 111 | -2.83 | 0.0056 |  | -2.1 | (-3.63, -0.57) | 104 | -2.64 | 0.0095 |
| IMT P1 * (5 months vs. BL) | -0.32 | (-2.91, 2.26) | 111 | -0.24 | 0.8081 |  | 0.02 | (-2.32, 11.22) | 104 | 0.01 | 0.9911 |
| IMT P1 * (12 months vs. BL) | 0.12 | (-2.49, 2.72) | 111 | 0.09 | 0.9305 |  | 0.08 | (-7.38, 8.50) | 104 | 0.06 | 0.9537 |
